# Non-cardiac Manifestations in Adult Patients With Mucopolysaccharidosis

**DOI:** 10.3389/fcvm.2022.839391

**Published:** 2022-03-07

**Authors:** Karolina M. Stepien, Andrew Bentley, Cliff Chen, M. Wahab Dhemech, Edward Gee, Peter Orton, Catherine Pringle, Jonathan Rajan, Ankur Saxena, Govind Tol, Chaitanya Gadepalli

**Affiliations:** ^1^Adult Inherited Metabolic Diseases, Salford Royal National Health Service Foundation Trust, Salford, United Kingdom; ^2^Northwest Ventilation Unit and Sleep Department, Wythenshawe Hospital, Manchester University National Health Service Foundation Trust, Manchester, United Kingdom; ^3^Academic Health Sciences Centre, University of Manchester, Manchester, United Kingdom; ^4^Intensive Care & Respiratory Medicine, Manchester University National Health Service Foundation Trust, Manchester, United Kingdom; ^5^Clinical Neuropsychology, Salford Royal National Health Service Foundation Trust, Salford, United Kingdom; ^6^Trauma and Orthopaedic Surgery, Salford Royal National Health Service Foundation Trust, Salford, United Kingdom; ^7^Neurosurgery, Salford Royal National Health Service Foundation Trust, Salford, United Kingdom; ^8^Manchester and Salford Pain Centre, Salford Royal National Health Service Foundation Trust, Salford, United Kingdom; ^9^Anaesthetics Department, Salford Royal National Health Service Foundation Trust, Salford, United Kingdom; ^10^Ear, Nose and Throat, Salford Royal National Health Service Foundation Trust, Salford, United Kingdom

**Keywords:** adult mucopolysaccharidosis, long-term complications, mortality, life span, long-term outcomes

## Abstract

Mucopolysaccharidoses (MPS) are a heterogeneous group of disorders that results in the absence or deficiency of lysosomal enzymes, leading to an inappropriate storage of glycosaminoglycans (GAGs) in various tissues of the body such as bones, cartilage, heart valves, arteries, upper airways, cornea, teeth, liver and nervous system. Clinical manifestations can become progressively exacerbated with age and affect their quality of life. Developments in advanced supportive treatment options such as enzyme replacement therapy (ERT), hematopoietic stem cell transplantation (HSCT) may have improved patients' life span. Adult MPS patients require specialist clinical surveillance long-term. In many cases, in addition to the MPS-related health problems, they may develop age-related complications. Considering the complexity of their clinical manifestations and lack of guidelines on the management of adult MPS disorders, multispecialty and multidisciplinary teams' care is essential to diagnose and treat health problems that are likely to be encountered. This review presents non-cardiac clinical manifestations, their pathophysiology, management and long-term outcomes in adult MPS patients.

## Introduction

Mucopolysaccharidoses (MPS) are a heterogeneous group of disorders (type I, II, III, IV, VI, and VII) that results in the absence or deficiency of lysosomal enzymes, leading to an inappropriate storage of glycosaminoglycans (GAGs) in various tissues of the body such as bones, cartilage, heart valves, arteries, upper airways, cornea, teeth, liver, and nervous system (1) (Table 1).

**Table 1:** Various types of MPS.

**MPS type (eponym)**	**Incidence per 10^**5**^ live births; inheritance pattern**	**Typical age at diagnosis**	**Typical life expectancy if untreated**	**Enzyme deficiency**	**GAG**
MPS I Hurler (H) MPS I Hurler-Scheie (H-S) MPS I Scheie (S)	0.11–1.67; AR	H: < 1 year H-S: 3–8 years S: 10–20 years	H: death in childhood H-S: death in teens or early adulthood S: normal to slightly reduced lifespan	α-L-iduronidase	DS, HS
MPS II (Hunter)	0.1–1.07; XR	1–2 years when rapidly progressing	Rapidly progressing: death < 15 years slowly progressing: death in adulthood	Iduronate-2-sulfatase	DS, HS
MPS III (Sanfilippo) A-B-C-D	0.39–1.89; AR	4–6 years	Death in puberty or early adulthood	Heparan sulfamidase (A) N-acetyl-α-D-glucosaminidase (B) acetyl-CoA-α-glucosaminidase N-acetyltransferase (C) N-acetylglucosamine-6-sulfatase (D)	HS
MPS IV (Morquio) A-B	0.15–0.47; AR	1–3 years	Death in childhood- middle age	N-acetylgalactosamine-6-sulfatase (A) β-galactosidase (B)	CS, KS (A) KS (B)
MPS VI (Maroteaux-Lamy)	0–0.38; AR	Rapidly progressing: 1–9 years slowly progressing: > 5 years	Rapidly progressing: death in 2nd-3rd decade slowly progressing: death in 4-5th decade	N-acetylgalactosamine-4-sulfatase	DS
MPS VII (Sly)	0–0.29; AR	Neonatal to adulthood	Death in infancy- 4th decade^**^	β-D-glucuronidase	CS, DS, HS
MPS IX (Natowicz)^*^	Unknown	Adolescence	Unknown	Hyaluronidase	CS

*Reproduced from Braunlin et al. (2), who complied data from Neufeld et al. (3) and Valayannopoulos et al.s (4)*.

*AR, autosomal recessive; CS, chondroitin sulfate; DS, dermatan sulfate; GAG, glycosaminoglycan; H, Hurler syndrome; HS, heparan sulfate; H-S, Hurler-Scheie syndrome; KS, keratan sulfate; S, Scheie syndrome; XR, X-linked recessive*.

* *Only 1 patient reported in literature (Natowicz et al. 1996)*;

** *death can occur in utero with hydrops fetalis*.

This results in alterations in cellular metabolism, which in turn leads to a range of manifestations that become progressively exacerbated with age. The MPS can affect multiple organ systems, affecting cognitive function and eventually resulting in severe debilitating and life limiting morbidity and premature death (5). Although they are multisystemic disorders, the affected individuals express phenotypic variability (5). All MPS types may have severe or attenuated presentations depending on the residual enzymatic activity of the patient. Very attenuated forms often present with atypical symptoms in adulthood. The improved longevity, increased disease awareness, early diagnosis of attenuated types has increased the number of patients with MPS who are 16-years-old and above.

Health-related quality of life (HRQoL) has been shown to be lower in patients with rare diseases, such as MPS, than in patients with other chronic diseases (6). Developments in advanced supportive treatment options such as enzyme replacement therapy (ERT), hematopoietic stem cell transplantation (HSCT) may have improved MPS patients' longevity and their HRQoL, including their performance in activities of daily living (6). The new upcoming therapies such as intrathecal or intracerebroventricular ERT and intravenous ERT with fusion proteins for neuronopathic MPS II seem to be able to reduce the levels of GAGs in the Central Nervous System (CNS) (7) and gene therapy and/or genome editing have shown promising results in preclinical studies (8). These therapies may reduce the impact of the neurological burden of the disease and improve clinical outcomes long-term.

The combined incidence of all types of MPS is about 1:22,000 live births (9). The incidence of MPS among adult patients is uncertain. Cardiorespiratory complications, including airway difficulties, are the main cause of mortality among MPS patients (10, 11). Given that in addition to the MPS-related health problems, adult patients may develop age-related complications which may not be directly related to their underlying metabolic condition, the overall risk of mortality is high. Therefore, adult MPS patients require specialist clinical surveillance in a specialist referral center life-long. Considering the complexity of their clinical manifestations and lack of guidelines on the management of adult MPS disorders, multispecialty and multidisciplinary teams' care is essential to diagnose and treat health problems that are likely to be encountered.

This review presents a detailed account of common non-cardiac clinical manifestations, their pathophysiology, management and long-term outcomes in adult MPS patients.

## Clinical Manifestations

### Upper and Central Airways

**Figure 1:**
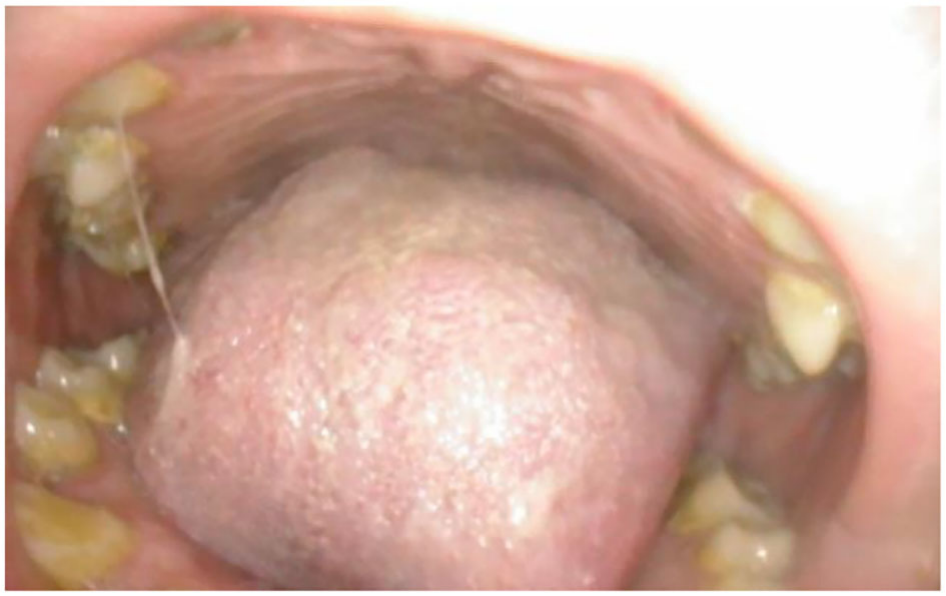
Oral cavity in MPSII; shows bulky tongue, small dysplastic teeth, mallampati grade IV, lack of curvature of the hard palate.

**Figure 2:**
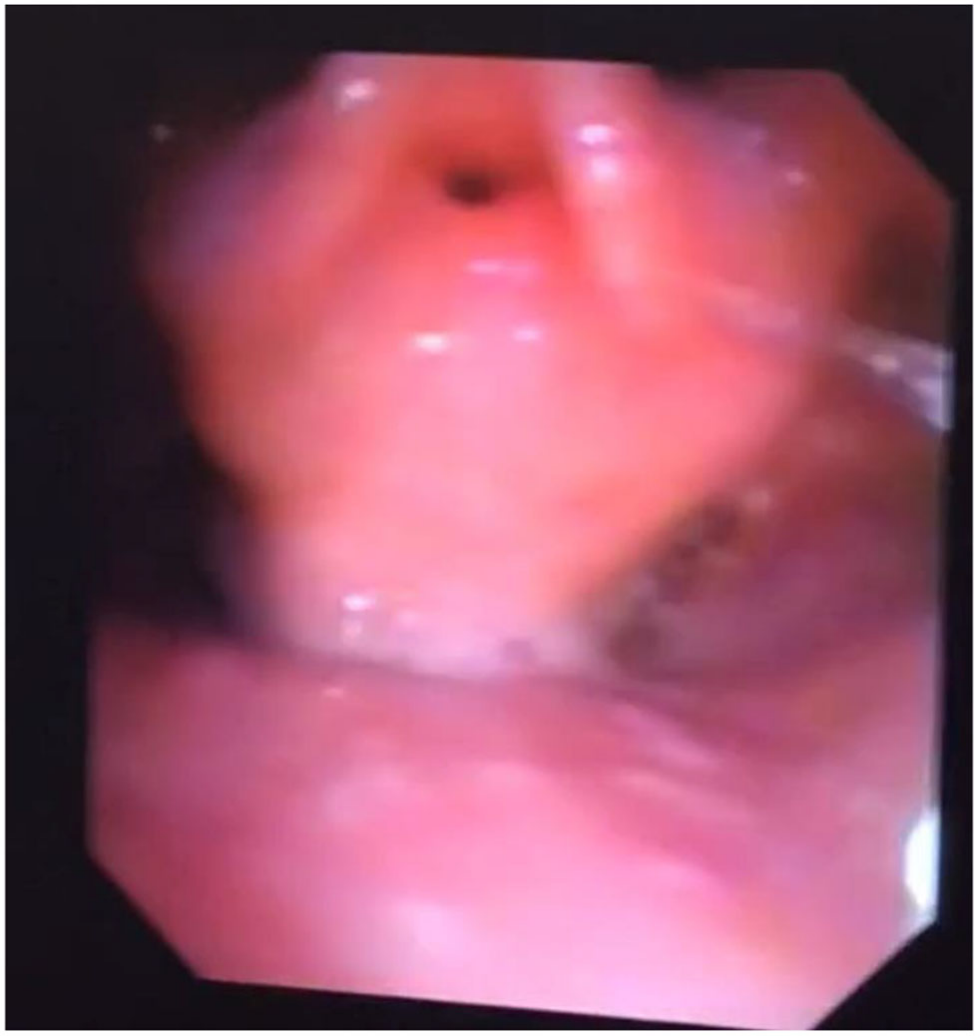
Nasendoscopy in MPS 2; bulky supraglottis and glottis with narrow glottic inlet.

**Figure 3:**
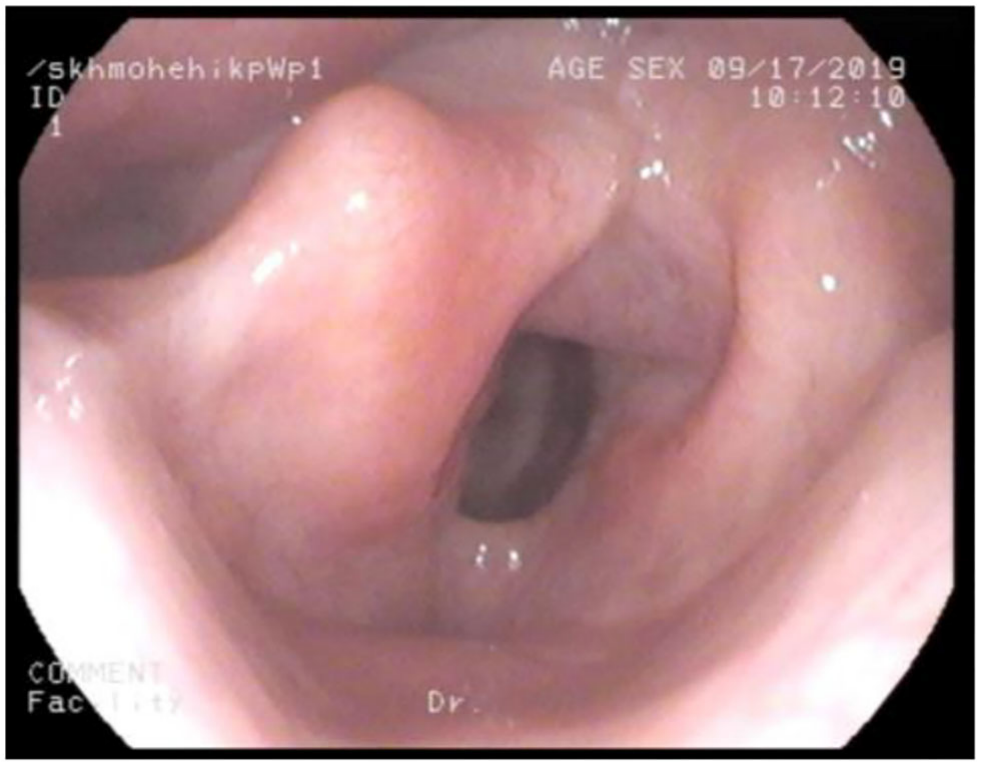
Nasendoscopy in MPS IV; large epiglottis and bulky anterior glottis.

**Figure 4:**
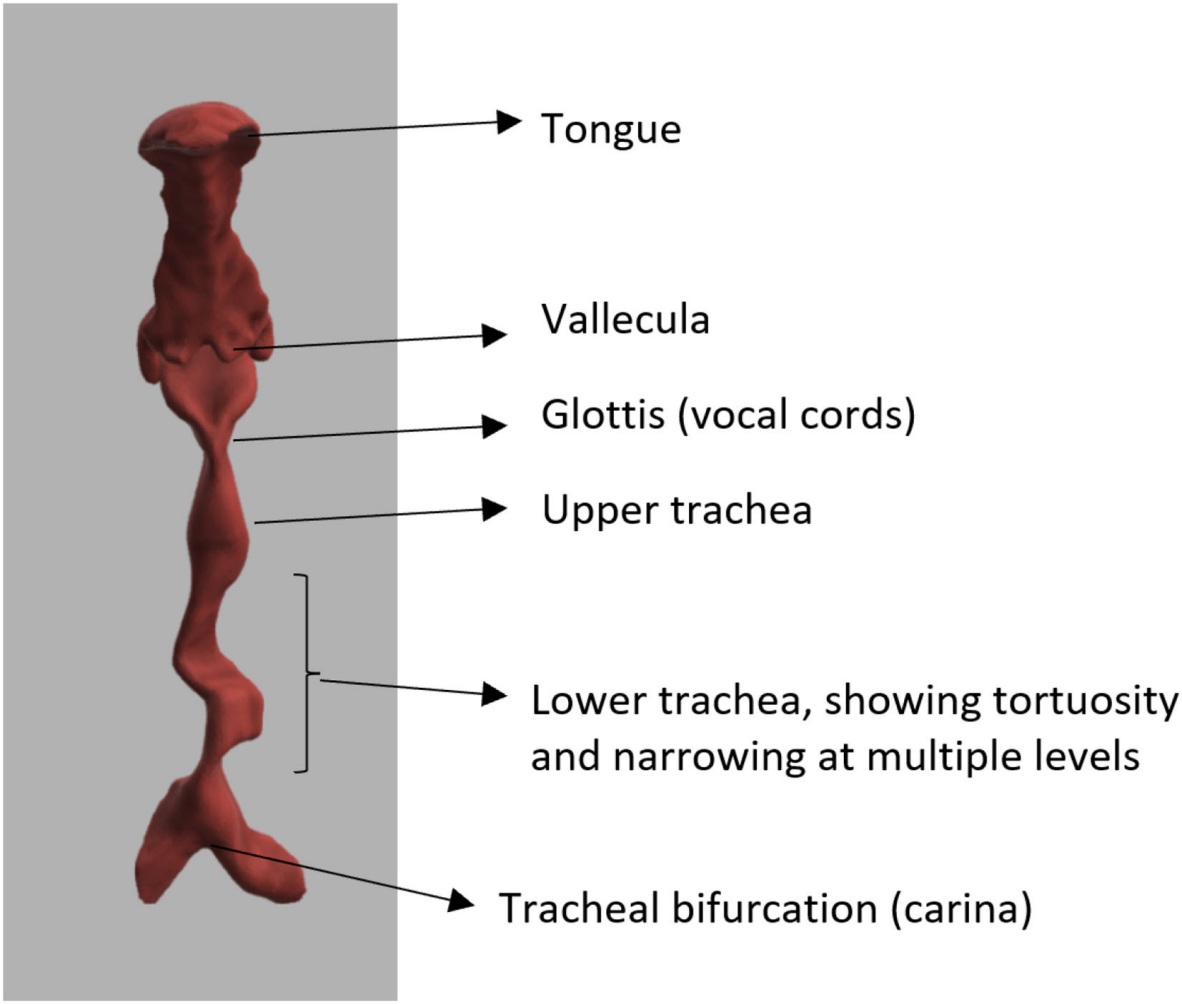
Three-dimensional reconstruction of airway in MPS VI, showing tortuous trachea.

Adult MPS patients have multisystem disease. The airway disorders are due to changes in the soft tissues and deformities in the skeletal system of the head, neck and chest regions due to deposition of GAGs. Knowledge of MPS airways is important as many of these patients will need some form of surgical intervention requiring anesthesia (12), the structural abnormalities and organ dysfunction can increase the risk of anesthetic complications (3). Knowledge of the MPS airways is also crucial as they may need airway intervention due to obstructive airway disease. Airway complications are a common feature of MPS I, II, IV, and VI and considerably contribute to morbidity and premature mortality (13, 14). In pediatric MPS, the predominant airway problems are obstructive airway disease and sleep apnoea from enlarged adenoids and tonsils. Adeno tonsillar hypertrophy is almost universal in MPS (15). As the child grows, the issue of adenoids and tonsils may have been addressed by the pediatric otolaryngologist. However, patients may need revision surgery later in adolescence and possibly adulthood (10, 16). Airway problems in MPS are multifactorial as evidenced by limited relief from adenotonsillectomy (14). The deposition of GAGs increases in the soft tissues and skeletal system leading to different problems in adult MPS. In these patients the airway problems move beyond adenoids, tonsils and moved toward the larynx, trachea and bronchi (15). The airway abnormalities could be varied such as infections, airway or respiratory compromise during or after anesthesia or sedation, breathlessness, obstructive lung disease, obstructive sleep apnoea, cor-pulmonale (17). Central sleep apnoea is possible due to spinal cord compression secondary to odontoid dysplasia and atlanto axial instability seen in MPS IV (18). The airway abnormalities in the upper or lower airways warrant holistic assessment by otolaryngologist, anesthetist, pulmonologist, and metabolic medicine. Similar to any adult complex airway, the assessment of adult MPS airway includes clinical history, examination, nasendoscopy, cross-section imaging and in some situations 3D-reconstruction of the airways. Figures 1–4 shows airway appearances in different MPS patients.

For ease of solving the problem of a complex airway, we can divide the issue into

1. Access to the airway- upper airway
  a. Guided by mouth opening, modified mallampati grade (19), cervical spine mobility, high or anterior larynx assessed either by thyromental distance (TMD) (20), nasendoscopy or existing cross-sectional images. A novel method has also devised to assess high or anterior larynx by measuring the distance between hyoid bone and mentum, hyomental distance and its inclination to horizontal, hyomental angle (21).
  b. Commonly used bedside airway assessments methods are neck movements, neck circumference, thyromental distance TMD (20), nasendoscopy, Wilson's score (22), mallampati (23), and modified mallampati grade (19). Based on laryngoscopy views Cormack (24) graded the airway into three grades; grade 1 being full view of the glottis, grade 2—partial view of the glottis, grade 3—only epiglottis is visible, grade 4—neither epiglottis nor glottis are visible. Various modifications to this (22, 25, 26) have been suggested. Most of the MPS I, II, IV, VI, VII have difficult laryngoscopy views (24).
2. Maintenance of the airway—central airway
  a. The glottis—MPS deposits can be noted on the glottis
  b. Subglottis size determines the size of the endo tracheal tube. The size of the tube is determined by age, sex, indication. As a general rule in adults, size 8 mm tube is chosen for women and size 9 for men (27) in the intensive care unit. However, this has to be carefully considered in short stature and smaller BMI individuals. An oversized tube can damage the laryngo-tracheal framework. The use of microcuffed tubes can be very useful as they cause little trauma to the airway mucosa and provide adequate ventilation.
  c. Varying degrees of tracheal narrowing and collapse have been noted in MPS (28). Tracheobronchomalacia can be severe and has been reported in adult MPS II (29).
  d. Tracheal tortuosity was observed in MPS IV, VI, and VII (30), this may be due to disproportionate growth of the spine and trachea. Surgical correction of the tortuous airways have been described as case reports (31).
3. Ventilation—peripheral airways
  a. Ventilation is determined by the caliber of the smaller airways and pulmonary tissues to facilitate gaseous exchange. Respiratory disorders are seen in all MPS types (13).
  b. MPS patients are known to have restrictive lung disease due to chest wall deformities leading to reduced compliance of the chest wall (14). The organomegaly from liver and spleen also splints the diaphragm reducing the chest compliance.

Airway assessment has to be holistic including various clinical, radiological and physiological methods (Table 2).

**Table 2:** Various methods for airway assessment.

**Method**	**Tools**
Clinical	History Examination Nasendoscopy Thyromental distance Hyomental angle Modified mallampati grade Prominent teeth Wilsons score Position of larynx and trachea in neck Cervical spine mobility/ stability
Radiological	Magnetic resonance imaging for upper airways Computer tomography (CT) scan for lower airways Dynamic expiratory CT scan for tracheo bronchomalacia
Invasive	Rigid or flexible endoscopy
Physiological	Pulmonary function tests Sleep study
Others	Three-dimensional reconstruction Virtual endoscopy Virtual reality

Based on all these methods a scoring system has been described for difficult airways, Salford Mucopolysacharridosis Airway Score (SMAS) (30). Appendix 1 provides the SMAS. The higher the score, the more difficult the airway system. It should be observed that each of the upper, central and lower airways have to holistically assessed to provide a complete airway assessment.

### Special Situations

#### Tracheostomy

This is a surgical procedure, where an opening is made on the anterior wall of the trachea to the skin of the neck to facilitate breathing directly into lungs and bypassing the upper airways. Tracheostomy has been found to be an effective way of managing upper airway obstruction in pediatric MPS II (32). Tracheostomy procedure may be surgically challenging in MPS patients due to poor access, short neck, lack of cervical spine extension. Following tracheostomy various tracheostomy tubes may have to tried to suit the best for the patient. Single lumen shiley^®^, bivona^®^ tubes montgomery t-tubes ^®^ can be useful. The use of t-tubes should be carefully considered as they are not easy to replace and knowledge amongst health professionals regarding t-tubes is poor. It becomes problematic if the patient have an obstruction in the t-tube. Following insertion of the tracheostomy, the tip of the tube may cause micro trauma to the trachea leading to GAGs deposits in the trachea.

#### Speech and Swallowing

It is important to consider speech and swallowing issues with MPS patients. MPS patients are known to have dental issues (33). The small dysplastic teeth do not allow them to properly masticate the food and swallow. The large tongue and bulky oropharynx may also not allow large amounts of food to be swallowed easily, lack of cervical spine mobility prevents laryngeal elevation. This act of laryngeal elevation is necessary for opening of cricopharynx to facilitate swallowing and prevent aspiration (34). In addition, some of these patients also have breathing difficulties, which can indirectly affect swallowing and speech. Voice in adult MPS patients is unique, it is usually low in pitch and appears strained. Most of the patients seem unaffected by their voice, however this may become a barrier in communication.

#### Communication

Vision, hearing, cognition are important aspects of effective communication. Ocular complications are common in MPS I and VI patients leading to poor vision (35). Wolfberg et al. (36) noted that hearing problems were common in MPS I, II, III, IVA, VI, and VII. MPS VI presents primarily with conductive hearing loss. MPS I, MPS II, MPS III, MPS IVA, and MPS VII can present with either conductive, sensorineural, or mixed hearing loss. The cause of sensorineural hearing loss remains unknown but it develops as the disease progresses. The effects of ERT on hearing function have been inconclusive. Reduced cognition due to learning disability may be noted in MPS I, II, III, VII; this can become a major barrier in treatment. Careful consideration to these disabilities has to be given and where possible alternative means of communication tools have to be established. Involving members of the family, establishing a partnership between patients and health professionals is vital.

### Airway Plan

It is important to have an airway plan for patients with MPS. This is because, MPS patients may need some form of surgical intervention under anesthetic, secondly, they may present with an airway problem as an emergency. All adult MPS patients have some form of airway abnormality (30). Having all the airway findings as a document or in electronic form is very helpful. Providing patients with difficult airway bracelets (37) are also helpful. The airway assessment should document the findings in upper, central and lower airways so as to plan the access, maintain the airway and ventilation. We recommend having the airway evaluation and an emergency plan in an electronic format, which can be possessed by the patient such as a pendrive, “the MPS passport” (38). Team working with the ENT surgeon, anesthetist, intensivist, and metabolic medicine are vital.

#### Intubation

Dose adjustments for all medications should written on a chart prior to the start of any intervention. Patients and family members should be allowed to be actively involved in decision making and a through communication with the patient and family members ensures effective patient- health professional partnership. For induction of anesthesia intravenous induction is preferable than inhalational, this is because of more control of the upper airway, blood pressure with intravenous anesthesia. Pre-oxygenation, Bi spectral Index (BIS) monitoring (39), intravenous cannulation, appropriate positioning should be done first. Supraglottic oxygenation with THRIVE (Trans nasal Humidified Rapid Insufflation, Ventilatory Exchange) (40) or high flow oxygen *via* a Nasopharyngeal airway is useful. Oxygen delivery with Nasopharyngeal airway has an additional advantage in preventing oropharyngeal, epiglottis collapse during induction. Laryngeal mask airway (LMA) use may be not be useful when the mouth opening is limited and in situations where supraglottis is bulky. Re-in forced LMA size 3 or 4 may be better than the bigger LMA. Should awake fiber optic nasal intubation be planned, adequacy of the nasal cavities and nasopharynx should be documented in the airway plan. Oral intubation following induction can be challenging. Use of video laryngoscope, Bonfils (41), pediatric bougie, hopkins telescope with a railroaded endotracheal tube is very useful. A small endotracheal tube such as microcuffed tube (42) may be useful, as it prevents mucosal trauma and accommodates well in the subglottis. In large bulky tongue, using a video laryngoscope from the corner of the mouth and pushing tongue completely to the left side provides more room to access the oropharynx and larynx. Spraying the larynx with 1:10,000 adrenaline and lignocaine prevents bleeding from mucosal trauma and laryngospasm. Should intubation not be possible, bag mask ventilation can be attempted with a guedel airway and nasopharyngeal airway in place. The guedel airway prevents the tongue collapsing in the pharynx and nasopharyngeal airway prevents epiglottis falling over the posterior pharyngeal wall. Airway scoring can be performed using SMAS (30). The indicators of difficult oral intubation include poor mouth opening, reduced cervical spine mobility, prominent teeth, high mallampati grade, high and anterior larynx shown on nasendoscopy and by calculating hyo-mental distance, hyo-mental angle (21). Blind passage of bougies and multiple airway intubation attempts should be avoided. Pre-planning airway and anticipating difficult airway are important to avoid a situation of cannot intubate, cannot ventilate. The difficult Airway Society, UK has provided guidelines (43) for management of airway and recommends that in an invent of cannot intubate and cannot ventilate, waking up the patient is the best option; however, if face mask ventilation is not possible, front of neck access such as cricothyroidotomy should be considered. The neck in MPS patients may be short and cervical flexion may be limited. Marking the cricothyroid membrane prior to intubation and having an ENT surgeon in theater is helpful.

## Respiratory Manifestations

Respiratory complications arising from both upper and lower airways and chest wall are common in adult patients with MPS. They can be amongst the first symptoms to present and become more prominent with age. All MPS types are associated with respiratory problems (13). The age of onset, prevalence, severity of symptoms, progress and prognosis varies between patients with MPS with different types. The management of MPS is continually evolving and patients continually survive into adulthood. We describe here the respiratory manifestations that can occur in adult MPS. The term adult MPS refers to MPS usually developing in childhood who survive to adulthood.

**Figure 5:**
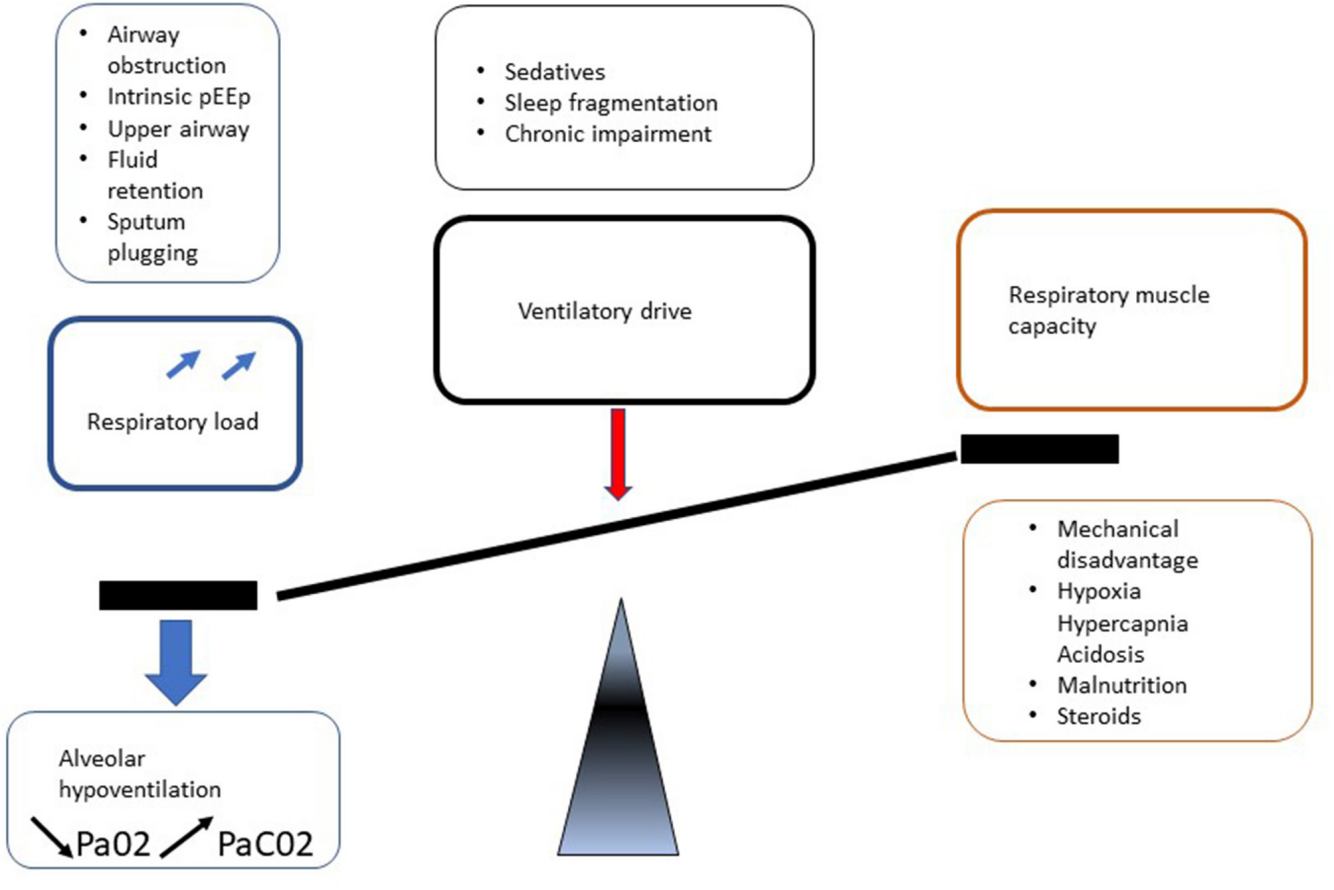
Mechanisms of respiratory involvement in adult MPS patients.

Respiratory manifestations of adult MPS result in different presentations depending on predisposing mechanisms such as upper and lower airway obstruction and restrictive pulmonary disease. In addition, involvement of MPS in central nervous system (CNS), spinal cord, spine, chest wall (kyphoscoliosis), and cardiovascular system leads to deterioration in respiratory function by a reduction in respiratory drive and diaphragm function, increase in airway obstruction and lung restriction (Figure 5). Berger and colleagues described this clearly (31).

In most MPS types, recurrent respiratory infections, upper and lower airway obstruction, tracheomalacia, restrictive lung disease, and sleep disturbances are common but to a varying degrees (14). For example, both upper and lower airway obstruction are prominent manifestations in MPS types I, II, and VI. Upper airway obstruction is usually more prominant than lower airway obstruction in MPS type IV. MPS types I and IV characteristically develop significant chest wall restriction.

**Figure 6:**
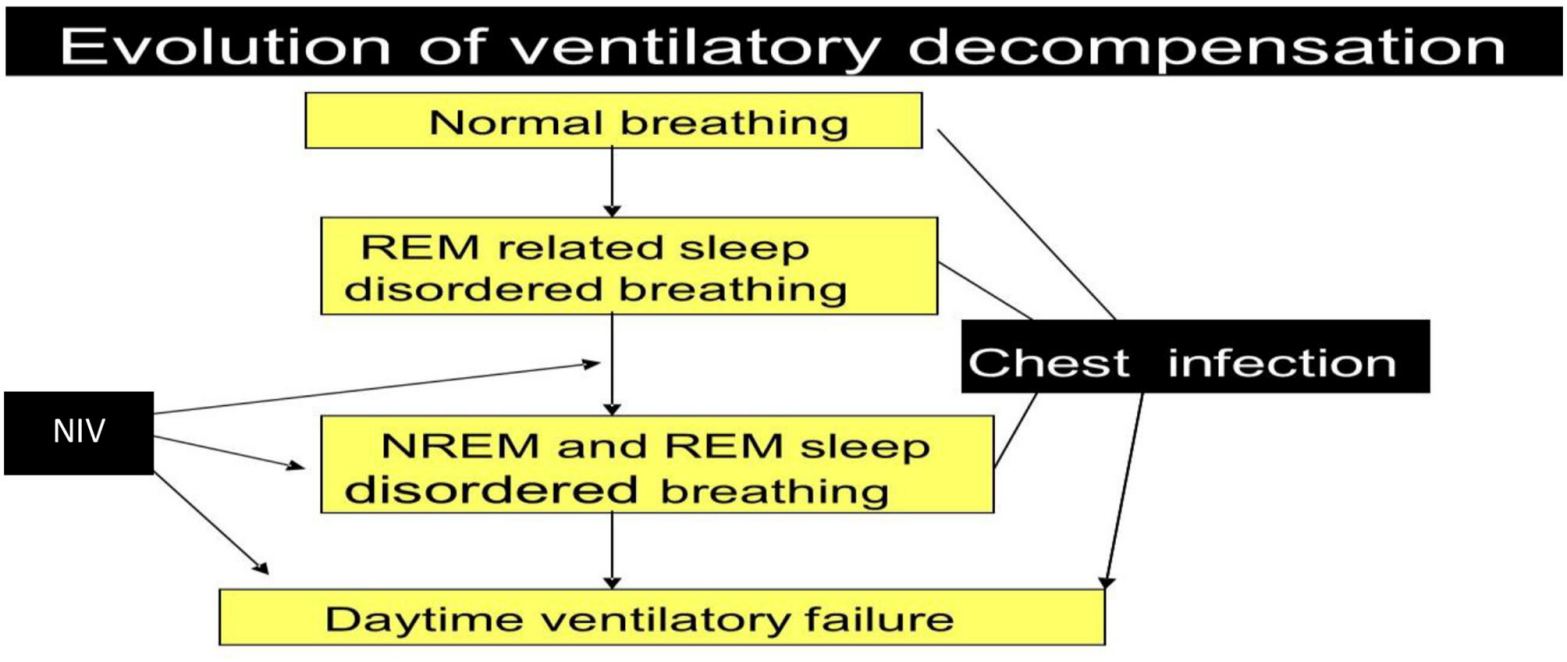
Progression of Sleep Disordered Breathing related hypoventilation in adult MPS patients.

Ventilatory failure as defined by impaired gas exchange progresses from nocturnal alveolar hypoventilation to daytime respiratory failure and cardiovascular compromise with cor pulmonale (Figure 6). When assessing the respiratory manifestations of adult MPS it is important to have an understanding of the physiology of respiration.

### Physiology of Respiration

Respiration results from the interaction of pathways under cerebral control. The brain controls the synchronous movement of the diaphragm, ribs and abdomen. There is modulation by feedback from arterial blood gas tensions, pH and pulmonary-mechanical factors. Chemical control is affected by the CO_2_ content of the blood. An increase in CO_2_ levels stimulates the respiratory center directly and also through the stimulation of carotid and aortic bodies. It is important to understand that the brain also controls phasic coordination of upper airway (UA) muscles, interacting on structural airway factors to determine the UA resistance (13).

### Anatomical Factors of Upper and Lower Airways

Structural abnormalities of both upper and lower airways are implicit in the development of respiratory complications and development of ventilatory failure. Unlike the lower conducting airways, the upper airway has no collateral ventilation. Supraglottic airway narrowing is common due to cranial and spinal abnormalities with features such as flattened nasal bridge, short neck, high epiglottis, mandibular abnormalities, abnormal cervical vertebrae, and GAG deposition in the mouth, nose and throat (13).

This can result in macroglossia, enlargement of adenoids and/or tonsils leading to collapse of pharyngeal and laryngeal walls. Excessive GAGs accumulation at the arytenoid cartilages and aryepiglottic folds can prolapse into the laryngeal inlet causing stridor and airway compromise (44). Airway obstruction in adult MPS is made worse by retention and inability to clear viscous secretions throughout the upper and lower respiratory system (45).

**Figure 7:**
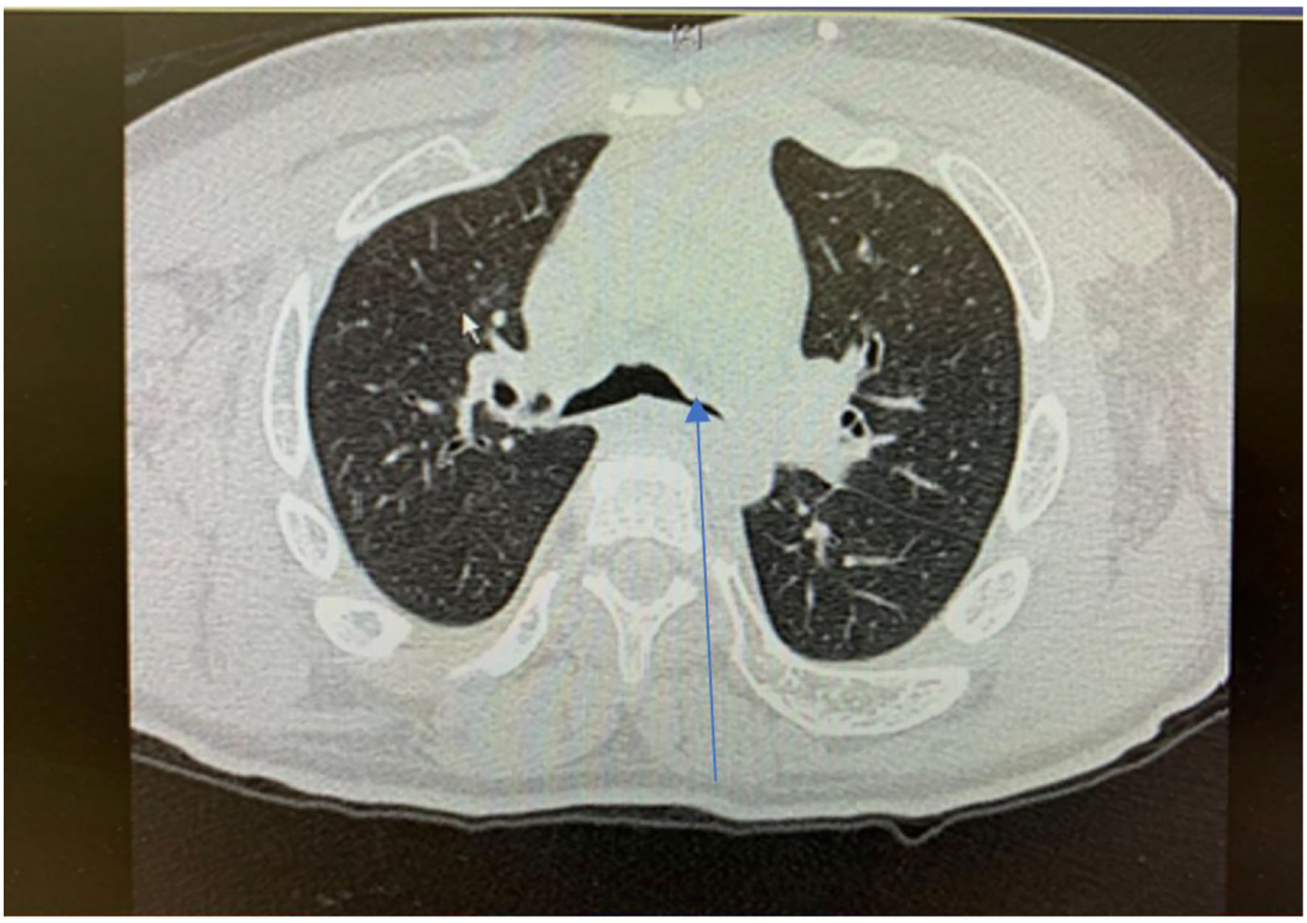
Cross sectional thoracic CT scan shows bronchomalacia in adult MPS IV.

Lower airway involvement presents as tracheobronchomalacia (TBM) and cartilaginous bronchi abnormalities develop secondary to GAGs accumulation in chondrocytes and extracellular matrix of trachea (46) (Figure 7). TBM manifests as collapse and narrowing of the trachea and cartilaginous bronchi. In addition, tracheal distortion is characteristic for MPS, especially MPS IV which reflects the disproportionate length of the trachea relative to the shortened spinal length (46). The degree of tracheal distortion can lead to collapse exacerbated by reduced tracheal traction as a result of small lung volume (47). In adult MPS II (Hunter Syndrome) patients, the large airways diameters are often strikingly reduced and upon expiration there is extensive collapse of the trachea and main bronchi, referred to as expiratory dynamic airway collapse (EDAC) (29). TBM of this degree contributes to the severe respiratory symptoms in patients with MPS II.

MPS IV patients may develop airway occlusion when flexing their necks. Characteristically they assume a “sniffing the morning air position” attempting neck extension to maintain airway patency. Consequences of tracheal and bronchial abnormalities include breathlessness, difficulty clearing secretions, cough, wheezing, and recurrent bronchitis or pneumonia. Upper respiratory hypersecretion/infections present in patients with MPS I, II, III, and VI from as early as the first months to 1 year of life (48).

**Figure 8:**
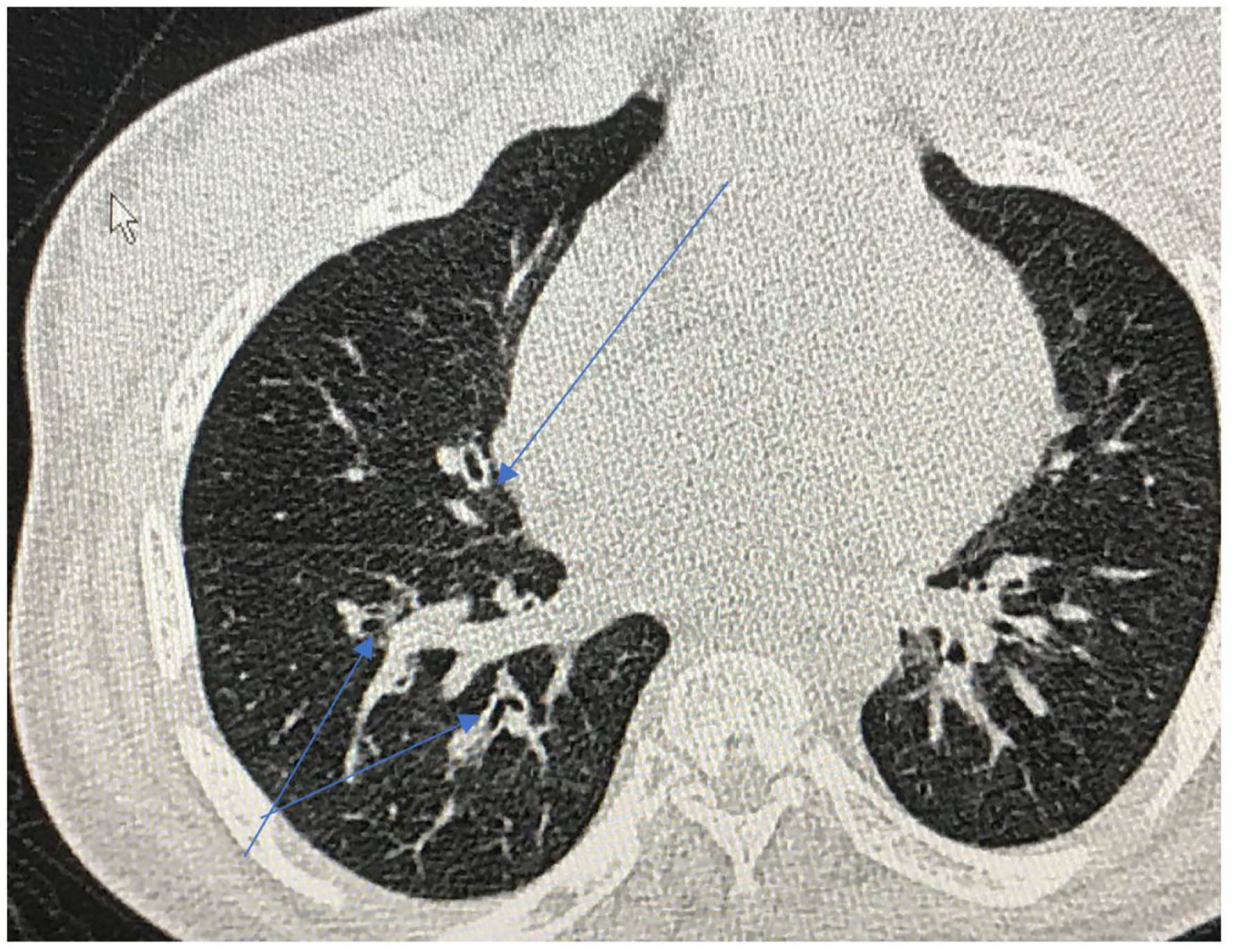
Cross sectional thoracic CT scan shows bronchial dilation and early bronchiectasis in adult MPS type IV.

Repeated infections, secondary retained secretions and inadequate clearance can lead to development of bronchiectasis (Figure 8). Development of multi-resistant organisms such as pseudomonas species is common among adult MPS patients.

Management strategies for clearing secretions include the use of mucolytics such as carbocisteine and, progressing to nebulized 7% hypertonic saline. The initial trial of nebulized 7% hypertonic saline should be supervised due to the risk of developing bronchospasm. This can be mitigated with prior use of nebulized salbutamol. Caution should also be taken with cough assist devices due to potential for exacerbation of airway collapse if a negative pressure expiratory cycle is used. Similar to long term management of other chronic obstructive pulmonary conditions, the role of long-term prophylactic antibiotics is recommended if repeated infections occur three or more times per year especially with evidence of bronchial wall thickening or bronchiectasis. As an example, azithromycin 250–500 mg three time weekly escalating to daily in the presence of frequent infections may be prescribed (49). Caution is exercised with prior checking of ECG to exclude a prolonged QTc interval and monitoring of liver function tests.

While ERT is effective in reducing urinary GAGs and liver and spleen volume in MPS I, MPS II, MPS VI, and MPS IVA, they have less impact on cartilaginous organs such as the trachea and bronchi, bones and eyes (50).

HSCT, however, has been shown to improve Sleep Disordered Breathing (SDB), including obstructive sleep apnoea syndrome (OSAS) in MPS IH, through reduction in adenoid hyperplasia, and reduced constriction from the tongue, and maxilla. The effectiveness may only be temporary, although airway management benefits during anesthesia using HSCT or ERT have been observed (51).

### Sleep Disordered Breathing (SDB) in Adult MPS

Alongside the respiratory complications seen in adult MPS SDB are a group of conditions which result in abnormal respiratory and ventilatory pattern during sleep. They include snoring, upper airways resistance syndrome (UARS), OSAS and central sleep apnoea (CSA).

#### OSA

Based on polysomnography analysis on a systematic review, the pre-treatment prevalence of OSA in MPS was 81% with a mean apnoea–hypopnea index (AHI) of 10.4 (52). Patients with MPS I are most significantly affected, with 75% suffering with moderate to severe OSA (mean AHI, 16.6). Obstructive sleep apnoea least affects MPS III. Apnoea index and apnoea-hypopnoea index were significantly higher in children than in adults with mucopolysaccharidoses (*p* = 0.03 and *p* = 0.03, respectively) (53).

Central apnoea is due to several factors in MPS patients, including spinal cord compression, raised intracranial pressure contributing to compression of the sleep regulatory centers in the brainstem, neurotransmitter alterations, and abnormal disrupted sleep cycle (54).

As described, SDB occurs in over 80% patients with MPS (although is more prominently seen in Types I, II, IV, and VI). This is as a result of increased upper airway resistance created by the skeletal, oral, adeno-tonsillar, laryngeal and tracheal involvement at multiple levels. It should be noted that the progression of sleep-disordered breathing to the development of ventilatory failure can have cardiac consequences, including cardio-respiratory failure, cor pulmonale and pulmonary hypertension (PH) (55).

Pulmonary Hypertension (PH) defined by a mean pulmonary arterial pressure (PAP) ≥25 mmHg; frequently complicates chronic lung disease (CLD) of various etiologies. CLD-associated PH (CLD-PH) has major consequences to patients with reduced functional ability, impaired quality of life, greater oxygen requirements and a higher mortality risk (56). Hypoxia and endothelial dysfunction play a central role in the development of PH. Cor pulmonale is a maladaptive response to PH. The presence of peripheral oedema in cor pulmonale, or right-sided heart failure, is almost invariably associated with hypercapnia (57).

Snoring, sleep disturbance, early morning headache, excessive daytime somnolence are the most common symptoms associated with SBD and disordered sleep architecture with OSAS. If not recognized early, patients may present late with PH and cor pulmonale which carries a worse prognosis if not managed correctly. Night sweats, bedwetting and behavioral changes including hyperactivity and aggressiveness may also be clinical features observed in children (58).

### Thorax

The thorax is composed of the bony rib cage, sternum, thoracic spine. Bony deformities, kypho-scoliosis can have an impact on the chest wall compliance. The common abnormalities noted are pectus carinatum, deformed vertebral bodies, short trunk (59). The splinting of the diaphragm can also me caused by organomegaly from liver and spleen. All these factors reduce the expansion of lungs.

### Chest Wall Restriction

As described multiple abnormalities in adult MPS patients can reduce ventilatory capacity, thereby manifesting as a reduction in vital capacity (VC). Chest wall restriction is most notable in MPS types I and IV. Kyphoscoliosis and pectus carinatum are common in patients with MPS and alter chest wall shape and structure. Lung function tests reveal a marked restrictive defect with reduced VC and high FEV1/FVC ratio. Many adult patients with MPS can struggle with performing of pulmonary functions tests but they can be helpful in determining degrees of obstruction vs. restriction, especially if a flow volume loop can be obtained. Diaphragm excursion may be compromised also by liver and spleen enlargement (45, 60, 61). Diaphragmatic impairment may theoretically result from spinal cord compression above the phrenic nerve origin (C3–C5), but there is currently no evidence for this hypothesis. Diaphragm function can be physiologically assessed by assessment of muscle pressures, maximum inspiratory pressure (MIP), maximum expiratory pressure (MEP) and sniff nasal inspiratory pressure (SNIP).

There is an interrelationship between sleep disordered breathing, chest wall and lower airway abnormalities contributing to the development of respiratory failure. Ventilatory compromise is exacerbated by normal sleep mechanisms that increase UA collapsibility thereby increasing the inspiratory effort demands. They are most prominent and occur initially during REM sleep, which causes loss of tone in the muscles exacerbating UA collapsibility. This leads to nocturnal alveolar hypoventilation, with hypoxia and hypercapnia and a reduction in ventilatory CO_2_ chemosensitivity (62) (Figure 6).

### Investigations and Treatment of Respiratory Complications in Adult MPS

The management of respiratory complications in patients with MPS is based on clinical presentation, anticipated condition progression and attempts to delay or prevent complications where possible. Screening procedures for SDB and development of respiratory failure such as overnight oximetry tests, transcutaneous CO_2_ monitoring are useful for assessment of hypoxia, hypercapnia, and hypoventilation. It should be noted that in the context of adult patients with MPS, symptoms of SDB (snoring, early morning headache, and daytime somnolence) may not predict the severity of the condition, thereby recommending regular screening, for example annually. They can be used to monitor progress and adjust treatment, especially importantly if the patient is receiving positive airway pressure (PAP).

Polysomnography and limited channels sleep respiratory studies remain the gold standard investigation for diagnosing SDB as they provide a more definitive diagnosis; and subsequently focused treatment; of sleep apnoea of obstructive, central, or mixed nature. Arterial/capillary blood gases analysis is essential when respiratory failure is suspected.

Continuous positive airway pressure (CPAP, Auto/Fixed pressure) therapy is the mainstay of treatment for OSA/UARS, that is not complicated with hypoventilation/hypercapnia or PH. It is initially delivered by mask interface overnight adjusted to correct SDB.

CPAP may also help as an alternative or adjunctive therapy to invasive procedures when treating tracheomalacia (TM). The positive airway pressure can act as a pneumatic stent, decreasing airway resistance and airflow obstruction (63). In infants with TM, CPAP increases maximal expiratory flow by raising Functional Residual Capacity (FRC) (64). The treatment of TBM in adults with MPS is complicated and success rates are variable because markers of success are often indirect, related to secretion clearance, frequency of lower respiratory infections in addition to more direct observations from high resolution CT scan imaging for example. TBM often progresses with age in MPS. Positive airway therapy can be delivered through a tracheostomy if necessary, for example due to upper airway deposits/obstruction.

Non-Invasive Ventilation (NIV) remains the choice of therapy in patients presenting with type 2 respiratory failure (both acute and chronic), central sleep apnoea and chest wall deformities, in the context of MPS respiratory related complications (65). It can also be indicated as an alternative option, for example, inadequate control of SDB through CPAP or when invasive/surgical procedures are deemed inappropriate. NIV refers to bilevel support with positive inspiratory pressure and a positive end- expiratory pressure. As with CPAP it can delivered by mask. If delivered through a tracheostomy it is then referred to as invasive ventilation.

It is essential that there is regular assessment for the development and monitoring of respiratory complications in MPS to identify the timing for intervention with either CPAP or NIV. Close liaison with ENT specialists is important because of the overlap and interaction with the development of both upper and lower airway complications. This is important for the decision-making process for consideration of a tracheostomy in an MPS patient who requires positive pressure ventilation.

Respiratory manifestations and complications in patients with different types of MPS can be among first signs and symptoms to present including SDB, upper and/or lower airway obstruction/collapse, TBM, chest wall deformities, recurrent infections and respiratory failure. The mainstay of the respiratory physician's armamentarium in the management of adult MPS is positive airways pressure (CPAP and NIV). It is a dynamic situation and many patients will progress from CPAP for SDB to NIV for either failure or disease pression to ventilatory failure with age. Early identification of TBM is essential and evaluation with inspiratory and expiratory CT scan images, pulmonary function testing and where possible bronchoscopy is important. The aim is to minimize the impact of TBM on airflow obstruction, secretion retention, recurrent infections and bronchiectasis. As with SDB and respiratory failure the lower airway complications related to chest wall restriction and TBM will also progress over time. Treatment options are limited but include the use of prophylactic antibiotics and chest clearance assisted techniques. Functional antibody status should be checked to advise pneumococcal vaccination and annual influenza vaccination should also be undertaken. Recurrent lower respiratory tract infections will lead to progression of complications, exacerbation of impaired secretion clearance and the vicious cycle of further infections, lung damage (bronchiectasis) and resistant organisms such as pseudomonas species (aeruginosa) (66). As respiratory and upper airway complications progress overtime CPAP and NIV may be less effective *via* a mask interface and consideration for ventilation *via* a tracheostomy should be considered.

As with any condition that leads to the development of respiratory failure, the respiratory manifestations of adult MPS can be life threatening. The types of MPS in adult patients most commonly effected with upper and lower airway complications (sleep disordered breathing, alveolar hypoventilation and TBM) include MPS I, II, IV, and VI and are most likely to progress into respiratory failure over time requiring positive pressure ventilation. Susceptibility to lower respiratory tract infections is increased especially in those with severe chest wall restriction such as MPS types I and IV. The main cause of death due to respiratory complications is respiratory failure secondary to mucus plugging and pneumonia.

## Neuropsychology

The impact of MPS in adulthood is not well-researched. Early neurocognitive and behavioral problems (67), coupled with the rarity and high mortality of MPS, means adult samples are small (68). The challenges of supporting adult MPS patients and their families are discussed.

### Neurocognition

MPS subtypes show varying degrees of CNS disease. MPS I, II, III, and VII are associated with global cognitive delay and decline (67, 69, 70) while MPS IV and MPS VI show neurocognitive impairments with or without progression (71, 72) (Table 3).

**Table 3:** Neuropsychological treatment outcomes in MPS.

	**Cognition**	**Behavior**	**Quality of life**	**Treatment outcomes**
MPS I	MPS IH: Significant neurocognitive impairments and decline (73) MPS IH/S & IS: Fewer neurocognitive symptoms (72)	MPS IH: Low self-esteem, depression and withdrawal in adolescence (67)	Pain, impaired living skills, and poor HR-QoL (74–76)	ERT: Improved neurocognitive function in MPS IH/S (77) Improved ADLs and HRQoL (74, 75) HSCT: Stabilized neurocognitive development (78, 79)
MPS II	Severe phenotypes demonstrate significant neurocognitive impairments (80, 81)	Hyperactivity, impulsivity, destructiveness, aggression, and sleep problems (82, 83)	Difficulty forming relationships, reduced ADLs, anxiety, and caregiver burden (84, 85)	ERT: Improved HRQoL in neurocognitively normal subjects (86) HSCT: Mixed results: Stabilization of neurological function in attenuated phenotype (87). Improved ADLs, but neurocognitive function remained unchanged or declined (88, 89)
MPS III	Significant neurocognitive impairments and decline (90)	Hyperactivity, impulsivity, destructiveness, and higher levels of aggression than other MPS subtypes, and sleep problems (83, 91)	Impairments in quality of life and significant caregiver burden (92)	ERT: Stable or decline in neurocognitive function in MPS IIIA (93) HSCT: Does not attenuate neurocognitive decline (94) SRT: No change in neurocognitive or behavioral deficits, or quality of life (95–97). Gene therapy: preliminary results in MPS IIIA show promise (98)
MPS IV	Neurocognitive impairment without progression (71)	Fewer behavioral and sleep issues, but can be fearful (83)	Pain, impaired ADLs and psychological difficulties and carer burden (68, 99)	ERT: Improved HR-QoL in MPS IVA (77)
MPS VI	Neurocognitive impairment without progression (72)		Significant impairments in ADLs (100)	ERT: Improved HR-QoL (101, 102)
MPS VII	Significant neurocognitive impairments and decline (69, 70)	Hyperactivity, challenging and repetitive behaviors, and lack of awareness of danger (70)		SCT: Limited data shows mixed results for neurocognitive function (69, 103)

*ERT, Enzyme replacement therapy; HSCT, Hematopoietic Stem Cell Transplantation; SRT, substrate reduction therapy; SCT, Stem Cell Transplant; ADLs, activities of daily living; HR-QoL, health-related quality of life; MPS IH, Hurler; MPS IH/S, Hurler-Scheie; MPS IS, Scheie*.

MPS IH (Hurler) demonstrates significant neurocognitive impairment, rapid progression, and early mortality, while attenuated phenotypes, like MPS IH/S (Hurler-Scheie), and MPS IS (Scheie), show less neurocognitive impairment (73). MPS III (Sanfilippo), and severe phenotypes of MPS II (Hunter) show significant delays in neurocognitive developmental (80, 90). Hearing loss, delayed language, and motor problems in MPS IH, MPS II (severe), and MPS III, further limit the acquisition of functional skills (81, 104).

Assessing neurocognitive changes in MPS disorders is crucial for supporting individuals and their families across the lifespan. However, accurate measurement is challenging (105). Many MPS cases score at “floor” levels on standardized psychometric instruments, and different test forms for pediatric and adult populations make it difficult to track progression over time. Neurocognitive evaluations are often important in Mental Capacity and Best Interest decisions (106), for example around consenting for surgical procedures and medical therapies, and neuropsychologists may be called upon to conduct such assessments and to support multidisciplinary decision-making.

Manifest as poor compliance in activities across home, educational, and occupational settings, neurocognitive problems are sometimes erroneously labeled as “disruptive.” Neuropsychological assessment and advice therefore play a crucial role in informing appropriate support plans to maximize functional outcomes. Neurorehabilitation strategies have been applied in MPS (107), but trials are lacking, and severe impairments will limit the effectiveness of standard patient-led strategies in adults. Rather, behavioral and environmental interventions [e.g., structured daily routines, minimizing distracting stimuli, adjusting communication to suit cognitive strengths, etc.] are more appropriate.

### Behavior

Behavioral issues may also be related to progressive neurological disease (82). MPS IH patients are seen as anxious, fearful, and compliant as children (83), but show low self-esteem, depression, and withdrawal when older (67) (Table 3). In MPS II and MPS III, hyperactivity, impulsivity, destructiveness, and aggression are reported, as well as sleep problems, including frequent waking, difficulty settling, and insomnia (83, 84, 92). MPS IIIA and IIIB show autistic-like behaviors in the form of social and affective problems (108). Difficulty in understanding the mental states of others (109) may impact on these issues. Fewer behavioral and sleep difficulties are reported in MPS IV (85). MPS VII patients demonstrate hyperactivity, challenging and repetitive behaviors, and lack of awareness of danger (70). In the presence of robust physical abilities, aggressive, and unpredictable behaviors are especially challenging for caregivers (83).

Standard neurobehavioural interventions (110) seek to modify antecedents to challenging behaviors, thereby reducing their frequency and severity. Antecedents may be biological (e.g., pain, fatigue, fragmented sleep), neuropsychological (e.g., neurocognitive impairment, anxiety, worry), and social (e.g., limited interpersonal skills). Situational antecedents (e.g., hospital visits, commencing new medical therapies, educational and occupational transitions, or family health issues) may also precipitate behavioral challenges. Poor social skills, such as inability to understand “normal” social rules, may lead to anxiety, low mood, social withdrawal, self-harm, and also raise the risk of exploitation from others, especially on social media sites.

### Quality of Life

Somatic and CNS burden in MPS cause pain, fatigue, and restricted joint mobility. This worsens problems in adaptive skills, school attendance, social and occupational engagement, low self-esteem and caregiver burden (111) (Table 3). Pain, impaired living skills, and poor HR-QoL are reported in MPS I (74–76). Carers and individuals with MPS II report high levels of anxiety, difficulty forming relationships, and reduced activities of daily living (ADLs) (84). MPS III impairs adaptive function and quality of life as the disease progresses (92). Psychological difficulties in MPS IV are related to pain and its impact on physical activities (99), and impairments in ADLs are seen in MPS VI (100), and MPS VII (70).

Caregiver burden is considerable across MPS subtypes (70, 112). In MPS II and MPS IVA, caregivers report a negative impact of disease progression on their physical and social functioning, emotional wellbeing, family relationships, daily activities, and finances (68, 86). Transitions from pediatric to adult care also affect psychological health and caregiver burden (113). Adult patients may feel anxious about the level of care they will receive in adult services, coupled with a sense of loss for those established relationships in the pediatric setting.

Adjunctive psychological and family therapies should be offered to adult patients and caregivers. Disease progression and uncertainty about the future make living with MPS feel like a never-ending cycle of loss and adjustment (92, 112), while a lack of knowledge and inconsistent professional support leave adult patients and their families feeling isolated after transition. Fostering trusted professional relationships is a crucial aspect of delivering quality care.

### Treatment

Most treatments fail to improve neurocognitive outcomes. ERT improves quality of life in various MPS subtypes (50, 87, 93) (Table 3), but is inefficient at crossing the blood-brain barrier, so does not alleviate neurological disease (114). Studies report stabilization of neurocognitive delays in MPS I after HSCT (78, 79), but mixed results in attenuated phenotypes of MPS II (87–89). No benefit of HSCT on neurocognitive function was noted for MPS III (94), and results are mixed for Bone Marrow Transplantation (BMT) in MPS VII (69, 103). Clinical trials of Genistein in MPS III report no change in neuropsychological, behavioral, or quality of life outcomes (95–97). Preliminary results in MPS IIIA show promise with gene therapy (98).

## Pain Management

Among different MPS disorders, type I, II, IV, and VI are characterized by spinal cord compression with neurological phenomena being typical of MPS I, II, III, and VII (38, 55, 115).

MPS patients experience a variety of different types of pain from nociceptive to neuropathic to mixed pains. Pain is a secondary consequence of GAGs deposition with different severity and characteristics of pain with different MPS subtypes (116). Skeletal deformity, muscle pain and the compression of visceral and neural structures, all contribute to pain in MPS patients (116).

### Epidemiology of Pain

Pain is a common feature across the spectrum of MPS patients with many authors having described pain in MPS and musculoskeletal issues being common in MPS I, II, III, IV, and VII patients (55, 115–120). Despite this pain is poorly recognized, assessed and treated with the incidence of pain being unclear and little known about the pathogenesis, epidemiology and treatment of pain in these conditions (116).

One reason for the dearth of data on pain in MPS may be due to the difficulties in its evaluation in the presence of cognitive impairment. Cognitive impairment occurs in MPS I, II and III with a higher pain prevalence having been reported in MPS patients with cognitive delay vs. those without (121). Musculoskeletal pain is common in MPS patients, particularly in MPS I, II, III, VI, and VI. Joint, back and hip pain are particularly prevalent (117, 121–123). Hendriksz et al. showed the joint pain is extremely common in MPS IV, with 64% of patients being affected (55).

Joint stiffness ranging from mild to severe is a hallmark of MPS aside from MPS IV and IX (122, 124). Joint stiffness may be due to GAGs storage in ligaments, tendons, and soft tissues as well as metaphyseal deformities (121, 122, 125). Skeletal disease including kyphoscoliosis and hip dysplasia which may contribute to pain continue despite ERT, as do genu valgum and joints contractures (126–130). Bone enlargement may contribute to joint swelling and stiffness (122). The upregulation of a neuropeptides including substance P in ligaments, menisci and periosteum and released from nociceptive free nerve endings may contribute to pain and lead to bone remodeling, and bone resorption (131).

The vascular system may contribute to pain as a result of vasospasm and ischemia. Coronary artery obstruction secondary to GAGs deposition in arterial lumens has been reported in MPS I in patients suffering from chest pain (132). The accumulation of GAG is in the liver and spleen, and consequent hepatosplenomegaly may lead to pain (55, 118, 122).

MPS patients suffer from neuropathic pain, including carpal tunnel syndrome secondary to GAGs deposition leading to compression of the median nerve. Patients may complain of pain with neuropathic features, such as burning and shooting and features of hyperalgesia and allodynia secondary to dysfunction of the nervous system. Patients may also suffer from spinal-cord injury with cervical stenosis and myelopathy due to ligamentous laxity, hyperplasia and GAG deposition. MPS IV in particular is characterized by cervical myelopathy and paraplegia which may contribute to pain (4).

Despite musculoskeletal manifestations being overshadowed by neurodegenerative manifestations in MPIII, a significant number of patients require evaluation for orthopedic issues with osteonecrosis of the femoral head and referred hip pain being reported (120). Vijay et al. investigated musculoskeletal problems in MPS and found a quarter had spinal deformities including kyphosis, scoliosis, and lordosis with carpal tunnel syndrome in half of patients and progressive arthropathy in 86%. However, pain itself was not investigated (118).

### Pathogenesis of Pain

The GAGs accumulation in bone cortex and a variety of other organs such as the brain, skeleton, kidney lung and liver may contribute to pain. In addition, inflammatory processes, alterations to gangliosides and lysosomal enzyme activity may contribute to the etiopathogenesis of pain (133).

CNS damage leading to alterations in dendritic cells with dendritogenesis, altered axonal morphology and alterations to synapses may occur due to altered glycosphingolipid storage. Accumulation of GM1 and GM3 gangliosides has been studied in MPS I, II, IIIA, IIIB, IIID, and MPS VI (134–136).

Overstimulation of monocytes/macrophages may lead to inflammatory cascades in MPS I and IIIB leading to central nervous system damage. Dermatan sulfate storage may also contribute to inflammation in MPS I, II, VI, and VII contributing to musculoskeletal problems. Dermatan sulfate may lead to the production of a chemical soup including pro inflammatory cytokines, proteases, nitric oxide and a pro apoptotic lipid ceramide (137, 138). This pro inflammatory milieu may then contribute to cartilage apoptosis, synovial hyperplasia and inflammatory joint destruction. Altered mineralization and osteoblast differentiation may also be caused by the action of Cathesin k on collagen ultimately driven by GAG deposition (139).

Tumor Necrosis Factor alpha (TNF-α) is has a fundamental role in pathogenesis of pain in MPS. Peripheral injury leads to the activation of PAMPS (Pattern Associated Molecular Patterns) and DAMPS (Damage Associated Molecular Patterns), leading to the activation of Nuclear Factor kB and Activator Protein 1 (AP1) *via* interaction with Toll Like Receptors including TLR4 and Nod like receptors (NLRs). This process in turn leads to activation of pro inflammatory cytokines, including TNF-α. This is also mediated by the P 38—MAPK system. TNF-α itself has been found to have a key role in the activation of other inflammatory cytokines including IL-1 beta, interleukin-6 and interleukin 8 (140). Neurons express receptors for such cytokines including TNF-α R. The activation of these receptors leads to the sensitization of nociceptive neurons contributing to peripheral sensitization (141–143).

The release of excitatory neurotransmitters at neurons can enhance dorsal horn signaling leading to central sensitization with widening of receptive fields, neuroplasticity and leading to the clinical manifestations of neuropathic pain, such as hyperalgesia.

Elevated TNF-α levels in MPS patients compared to healthy controls are associated with increased pain and decreased physical function despite ERT or HSCT. Effects of TNF-α include those on matrix metaloproteinases (MMP), prostaglandins and inflammatory cells leading to destructive lesions. As such TNF-α antagonist may have a role to play in ameliorating pain. Indeed, anti-TNF-α medications have shown improvements in exercise, joint destruction and bone length (137, 144, 145).

The role of glial cells in pain in MPS must also be considered. Glial cells may confer trophic support to neurons in the CNS. It is likely that GAG deposition leads to destruction of lysosomes with the release of heparan sulfate and other proteases acting as DAMPS, which activate microglia. Microglia sense DAMPS *via* TLR4 (146). Thus in chronic inflammation glial cells can contribute to neuroinflammation as a downstream effect of GAG deposition and in a feed forward cycle of nociception mediated by nkkb and the p38MAPK system (147). Furthermore, excess GAG deposition may lead to activation of TLR-4 triggering TNF-α production and prostaglandin E2, leading to pain and osteopenia, inflammation and macrophage activation (148).

The migration of macrophages to other sites distort from injury recruited by this pro-inflammatory milieu likely also contribute to maintenance of pain. In the future, non-neuronal targets for controlling pain targeting TNF-α, the TNF-α R, IL-10, and IL-4 may be key in combating pain.

### Evaluation of Pain

Evaluation of pain in MPS requires that nociceptive, neuropathic acute and chronic pain are measured as well as qualify life scales. This poses challenges in the pediatric population where the FLACC (Face, Legs, Activity, Cry, Consolability) scale has been scale of choice. The revised FLACC scale may be more suitable in patients with cognitive impairment (149). In the adult population a variety of scales are used but have not been validated in the MPS cohort. They may be classified into globalized pain scores, neuropathic pain scores and quality of life scores as in Table 4. The presence of cognitive impairment impacts on utility of such scales, in the adult population. A wide variety of pain related outcomes are used in MPS including the pediatric pain tool, six faces scale revised for patients older than 8 years old; the non-communicating children pain checklist revised for children aged 3–18 with cognitive impairment, and the Achenbach scales for social and adaptive psychological issues. Function based patient reported outcomes have been developed and the Childhood Health Assessment Questionnaire and Health Assessment Questionnaire for adults have been used to measure pain in ERT based trials (150, 151). Few patient related outcomes related to fatigue. MPS patients often have elevated levels of anxiety and distress and psychological issues including anxiety and depression. The correlation between pain and psychosocial distress further highlights the need for holistic care as well as analgesic therapies and therapy for the underlying disease process. However, the presence of cognitive decline may hinder assessment of pain and related distress of both parents and carers (85, 99, 111).

**Table 4:** Measures used in evaluating pain and quality of life in MPS patients.

**Symptom**	**Scale**	**Domain measured**	**Notes**	**Cardiac caveats**	**Renal caveats**
Neuropathic pain	Leeds Assessment of Neuropathic Pain Sign and Symptoms Scale	Neuropathic pain	Pain of predominantly neuropathic origin if score >12. Not suitable with sever cognitive impairment.		Reduce dose in renal impairment
	Pain Detect	Neuropathic pain		Caution: arrhythmogenic Monitor QTc interval.	Reduced dose if eGFR <30 Reduce dose in renal impairment
Global pain scores	Faces Scale	Global Measure of Pain	Pediatric Population and in cognitive impairment Use of visual scale.		None
	FLACC, r-FLACC face, legs, arms, cries, consolability scale	Global Measure of Pain	Utility in cognitive impairment. Carer administered.		
	Visual Analog Scale	Global Measure of Pain	Weight gain, cognitive dysfunction, lethologica		Reduce dose in graduated fashion with renal impairment
	Abbey Pain Scale	Global Measure of Pain	In those with cognitive impairment and elderly.		Reduce dose in renal impairment
**Quality of life scores**					
	Brief Pain Inventory	Measure of Pain impact on function, mood, sleep, interactions with people, analgesic response,	Informs holist care measures limited utility in severe cognitive impairment and limited with visual impairment		None
	Patient Health Questionnaire Nine	Measure of Depression	Informs holist care measures limited utility in severe cognitive impairment and limited with visual impairment.		None
	Generalized Anxiety Disorder 7	Measure of Anxiety	Informs holistic care measures. Limited utility in severe cognitive impairment and limited with visual impairment		
	Tampa Scale of Kinseiophobia	Measure of Kinseiophobia	Informs holistic care measures. Limited utility in severe cognitive impairment and limited with visual impairment		
	Patient Catastrophisation Scale	Measure of Catastrophizing	Informs holistic care measures. Limited utility in severe cognitive impairment and limited with visual impairment		

### Treatment Options

Treatment of pain involves analgesia and the treatment of the disease process itself. Treatment of the disease in MPS I, IV, and VI has shown to lead to pain reduction and improve quality of life. MPS I, II, IV, VI, and VIII may be treated with ERT. However, whilst it may attenuate symptoms it does not prevent disease progression (152). Laronidase for MPS I has improved walking distance and shoulder mobility whilst Gasulfase for MPS VI has shown to lead to decrease in pain from baseline (150, 151). Furthermore ERT does not cross the blood brain barrier with bones, eyes and the respiratory system poorly penetrated (50).

The TNF-α inhibitor, adalimumab has been trialed in MPS I and II patients where increased TNF-α levels are thought to be central to joint destruction and are associated with pain and dysfunction (126, 153). It has been well-tolerated without serious adverse sequalae. Bodily pain scores measured using the CHQ-PF50 reflected lower levels of pain vs. placebo, whilst range of motion improved marginally in a variety of joints, there was no improvement in pediatric pain questionnaire metrics, 6-minute walk test or hand dynometer function (153). This suggests Adalimumab and other TNF-α inhibitors may have a role in improving pain, quality of life and reducing joint destruction in future after further clinical trials (126).

MPS patients are subjected to multiple surgeries throughout life and as such experience episodes of acute and acute on chronic pain during the perioperative period (154, 155). Acute pain may be more challenging to manage due to cognitive issues, short stature, raised intercranial pressure and spinal abnormalities impossible impacting upon analgesic techniques and the wider experience of adult interdisciplinary teams (113).

Furthermore, as patients transition from pediatric to adult care individualized care plans are of great importance. Traditional holistic management may be impacted by the impaired cognition and behavioral symptoms associated with GAG deposition progress during adulthood (115, 156, 157). Analgesics may impair cognitive function leading to further challenges in acute pain management. Patients also face age-related pain related as other non-MPS patients would. However, due to cognitive decline and impaired renal and rehabilitative function, these age-related phenomena and their knock-on consequences may be harder to treat using traditional medicine and treatments.

Table 5 summarizes the variety of pharmacological and non-pharmacological modalities of treatment of pain in MPS disorders. In addition, surgical procedures for hip dysplasia, severs scoliosis and carpal tunnel syndrome may aid pain relief in MPS patients.

**Table 5:** Non-surgical pain management modalities in MPS.

**Agent**	**Mechanism of action**	**Dose**	**Side Effects and cautions**	**Cardiac caveats**	**Renal caveats**
**Neuropathic pain**					
Tricyclic antidepressant - Amitriptyline - Nortriptyline	5HT and NA reuptake inhibition Action on Dopaminergic pathways and locus coeruleus	12.5–150 mg/day	Dry mouth, sedation, arrythmias, urinary retention Diarrhea, cognitive disturbance, worsening of autonomic instability		Reduce dose in renal impairment
Serotonin and noradrenaline reuptake inhibitors - Duloxetine - Venlafaxine	5HT and NA reuptake inhibition	60–120 mg/day 150–225 mg/day	Serotonergic syndrome Gastrointestinal discomfort diarrhea, anxiety, dizziness,	Caution: arrhythmogenic Monitor QTc interval.	Reduced dose if eGFR <30 Reduce dose in renal impairment
Carbamazepine	Reduced Na + channel conductance. Reduction in ectopic discharges	250–800 mg/day in two divided doses	Associated with blood dyscrasias, Steven's Johnson's syndrome, Toxic Epidermal necrolysis and hyponatraemia		None
Gabapentin	Inhibit calcium mediated neurotransmitter release through effects on *α _2_ δ-*1 subunits. NMDA Receptor antagonism	Titrated from 100 mg/day to 3,600 mg/day in three divided doses.	Weight gain, cognitive dysfunction, lethologica		Reduce dose in graduated fashion with renal impairment
Pregabalin	As gabapentin	Starting dose 50 mg bd up to 300 mg bd	As gabapentin		Reduce dose in renal impairment
**Nociceptive and acute pain**					
Paracetamol	Local anesthetic causing sodium channel blockade	2–5 mg/kg			None
Non-Steroidal Anti-inflammatory Drugs Ibuprofen, Ketoprofen, Naproxen, Ketorolac, Indomethacin	Inhibition of Prostaglandin synthesis		Burning, pruritus		Reductions needed in severe renal impairment
Tramadol	Noradrenaline and serotonin reuptake inhibitor. Mu (μ) opioid receptor agonist.	100–400 mg/day	May lower seizure threshold		Caution in renal insufficiency
Codeine		Dosing according to weight	Nausea, constipation, itch, respiratory depression, osteoporosis, reduced immunity, endocrine dysfunction.		Caution in renal insufficiency
Morphine			*		Caution in renal insufficiency
Fentanyl			*		Caution in renal insufficiency
Oxycodone			*		Caution in renal insufficiency
**Adjunctive measures**					
Bisphosphonates			Caution in Renal Insuffiency.		
Glucocorticoids	Treatment of Hepatic distension				
Cannabinoids	Stimulation of CB1 and CB2 receptors, action on serotoninergic receptors.		Decreased appetite, nausea, vomiting, fatigue, mood changes, suicidal ideation. Few good trials		
**Physical therapies**					
TENS	Mechanism on A beta fires leading to “gating” of nociceptive input.				
**Psychological therapies**					
	Distraction				
	Hypnosis				

Physical and occupational therapy form the basis of rehabilitation and may enable children to attain developmental milestones. Physical therapy can also be used for kyphoscoliosis occurs between 18° and 45° to avoid neurological problems and reduce pain (158–162). Physical therapy can also be a benefit in hip dysplasia to strengthen hypertonic muscles and reduce pelvic instability and asymmetry (163). Hydrotherapy reduces joint loading and improve strength and can be used for patients with motor disabilities with MPS one and two (11, 164, 165). Hydrotherapy affords buoyancy to support joints with increased neurosensory feedback compare to land exercises (166). Hydrotherapy cannot be considered in adult MPS patients who have tracheostomy. Non-surgical techniques such as radiofrequency ablation for hip and knee pain may provide an alternative for patients suffering with hip dysplasia and genu valgum in whom surgery and anesthesia is challenging and high risk (166, 167).

The correlation between pain and psychological distress further highlights the need for holistic care as well as analgesic therapies and therapy for the underlying disease process. However, the presence of cognitive decline may hinder assessment of pain and related distress of both parents and carers (85, 99, 111).

## Foot and Ankle Abnormalities

Adult patients with MPS are entering the sphere of the orthopedic surgeon because of persistent musculoskeletal manifestations in conjunction with the success of medical advances in systemic treatments contributing to prolonged life and better functional and cognitive development (168).

The condition is characterized by the accumulation of GAGs in the tissues, with effects on chondrocytes, extracellular matrix, articular cartilage and synovium (138). Pro-inflammatory cytokines (IL-1β and TNF-α) are induced by the presence of GAGs in cartilage and synovium (138). The result is increased osteoclast activity, abnormal synovial proliferation and pathological cartilage apoptosis through the dysregulation in normal cell turnover (138). Hyperplasia and hypertrophy have been observed in MPS synovial lining cells which would be consistent with IL-1β, TNF-α and LPS signaling (138, 169, 170). This pathological process can explain the joint stiffness and contractures in tendons, ligaments and the capsule surrounding joints (171). Elevated levels of TNF-α also increase synthesis of Matrix Metalloproteinases (MMPs) in MPS synovial membranes, accelerating articular cartilage degradation (138, 172, 173).

Bony dysplasia is another common finding and is multi-factorial. Elevation of Receptor Activator of Nuclear factor Kappa- β Ligand (RANKL) in bone marrow and synovial tissues (138) (caused by IL- β1 and TNF-α) increases osteoclast differentiation resulting in pathological bone resorption and osteopaenia (173). Failure of chondrocyte maturation limits longitudinal physeal growth, contributing to short stature (171), and decreased longitudinal septae in the metaphyses leads to abnormality in metaphyseal shape (12). These manifestations apply altered mechanical forces on a poorly organized physis, leading to secondary skeletal dysplasias during growth.

Musculoskeletal effects can be mild or severe and may present with direct or indirect etiologies. Direct manifestations include skeletal dysplasia (3, 171, 173–175) avascular necrosis of the epiphysis (12, 176, 177) and loss of compliance of the joint capsule (178). Indirect manifestations include loss of muscular compliance due to GAG deposition, causing imbalance of muscular forces, and spinal nerve root compression causing muscular spasticity or flaccidity (61). ERT and HSCT are effective systemic treatments but deliver poor extracellular enzymatic penetration at osseo-cartilaginous tissues (12, 119) allowing musculoskeletal manifestations to progress.

There is no literature at present that explores foot and ankle deformity along with its management in adulthood, but patients are likely to present with progression of childhood presentations. Symptoms can include painful, stiff and/or hypermobile joints, instability, swelling caused by underlying bone deformity, and gait abnormalities such as toe walking caused by contractures at the ankle and Achilles tendon (171, 177). Various foot and ankle deformities have been described across the MPS spectrum including pes planus, ankle valgus deformity, distal tibial epiphyseal wedging, shortening of the fibula, equinus contractures and cavovarus deformity (119, 178, 179).

In MPS I (Hurler's syndrome) foot and ankle deformities include pes planus, hindfoot valgus, curly toes and Tarsal Tunnel Syndrome (TTS) (180, 181). Ankle valgus is likely a consequence of distal tibial epiphyseal dysplasia causing primary bony deformity and a secondary effect of genu valgum (valgus at the knee) placing valgus mechanical force on the physes (leading to abnormal growth *via* Heuter Volkmanns law where supraphysiological forces inhibit longitudinal physeal growth) and soft tissue constraints (causing progressive valgus of the subtalar and ankle joints and flat foot collapse) (180). GAG deposition in the flexor tendons, retinacula, joint capsular tissues and muscles (182) around the ankle joint [also demonstrated in the wrists and hands of patients with MPS I (183)] will cause soft tissue stiffness and loss of muscle and tendon compliance leading to reduced range of ankle motion. Hindfoot valgus places the posterior tibial nerve (on the medial side of the ankle) on increased stretch and GAG deposition is seen in the tarsal tunnel, both can cause symptoms and signs of TTS that include numbness, tingling and pain in the ankle or sole of the foot (181). Spinal conditions such as cervical stenosis, thoraco-lumbar kyphosis, and severe scoliosis are common in MPSI and, as such, nerve root and spinal cord compression should be excluded first.

**Figure 9:**
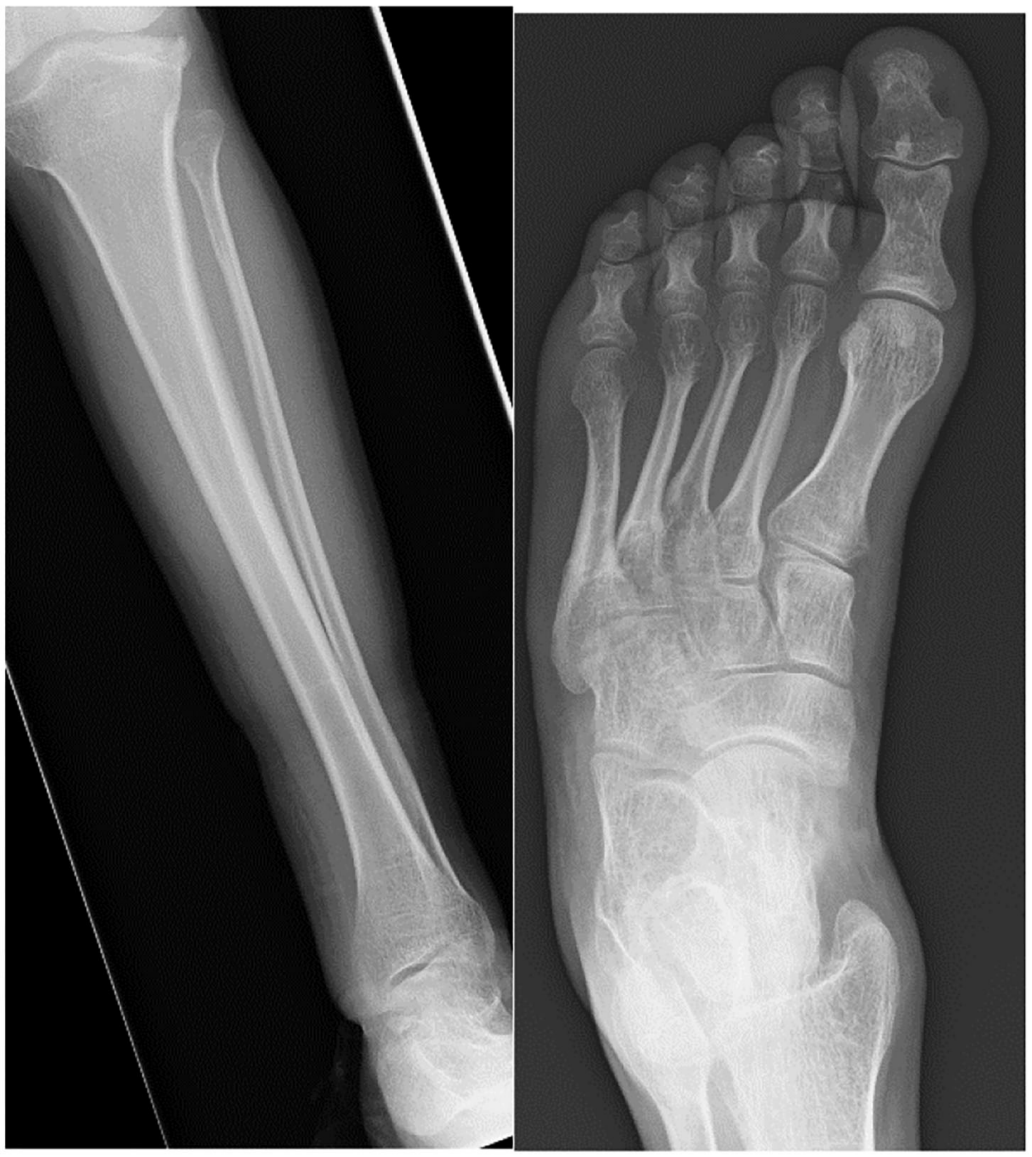
AP radiographs of the tibia and fibula and an AP view of the foot in a 22- year-old male with MPS I. The tibia shows epiphyseal dysplasia on the lateral side of the ankle and fibula hypoplasia causing valgus wedging with a decreased LDTA, tipping the hindfoot into valgus. The foot shows dysplastic and shortened metatarsals with widened proximal metaphyses.

Kennedy et al. (180) describe foot and ankle pathologies in 18 MPSI patients (mean age 10.3 years). Ankle valgus was present in 13 patients evaluated by weight bearing radiographs. The average lateral distal tibial angle (LDTA) was 70.6° (normal range 86°-93°), lateral talo-calcaneal (TC) angle was 45.8 degrees, antero-posterior TC angle 31° and talus first metatarsal angle 10.7°. Morphological examination revealed moderate to severe pronation in 21/36 feet, 6 had flexible deformity, with the remaining 15 not correcting when standing on tiptoes. The older children tended to have a more rigid deformity, which could be explained by progressive deposition of GAGs in the soft tissues. Curly toes were also present in 13/18 patients and these are suggestive of soft tissue tightness from muscular contractures, spasticity, valgus of the ankle increasing flexor tendon excursion or soft tissue contractures. Functional outcome scores revealed 14/18 had significantly limited function of the foot and ankle. Williams et al. (181) reported three MPSI patients with TTS undergoing tarsal tunnel decompression, 2 of which had good results (181, 184). Figure 9 demonstrates the radiological changes seen in an adult patient with MPSI.

MPS II (Hunter's Syndrome) is associated with joint stiffness (185). Musculoskeletal manifestations are dominated by spine conditions (cervical stenosis, thoracolumbar kyphosis and scoliosis), hip conditions (hip dysplasia), and carpal tunnel syndrome (119, 185). There are no reported cases of foot and ankle pathologies in MPS II however given the propensity for carpal tunnel syndrome in this condition, which can present with thenar muscle wasting, night pain in the hands and numbness/tingling in the lateral three and a half fingers, future case series may reveal TTS is caused by a similar etiology.

Musculoskeletal manifestations in MPS III (Sanfillipo syndrome) are generally less severe than other forms of MPS (177, 179). Typically, symptoms are from the central nervous system as a consequence of scoliosis, kyphosis and hypoplasia (179) with adolescent loss of motor and cognitive abilities (169). Bony deformity is absent or mild. At the ankle, equinus and equino-varus contractures are described (177) and may be due to contractures of the joint capsule, tendons and synovial tissues (171, 177, 179) or spinal deformities causing nerve root compression, leading to imbalanced muscular spasticity or flaccidity. Split posterior tibial tendon transfer with tendoachilles lengthening, and plantar fascia release for one child with equinovarus deformity was performed with no immediate complications (177).

MPS IV (Morquio-Brailsford Syndrome) is characterized by ligamentous laxity and joint hypermobility compared to the other MPS types where joint stiffness predominates (178, 186). Metaphyseal deformity, bony hypoplasia and ligamentous laxity (187). lead to an inability to maintain a favorable mechanical axis, causing progressive deformity at the hips, knees and ankles. Ankle valgus, pes planus and external tibial torsion cause an externally rotated foot with a valgus ankle, midfoot collapse, soft tissue laxity and muscular weakness all leading to poor power generation during the terminal stance phase or “push off” (171, 186). Avascular necrosis and collapse is also a common finding and can affect multiple bones of the foot and ankle causing significant pain.

**Figure 10:**
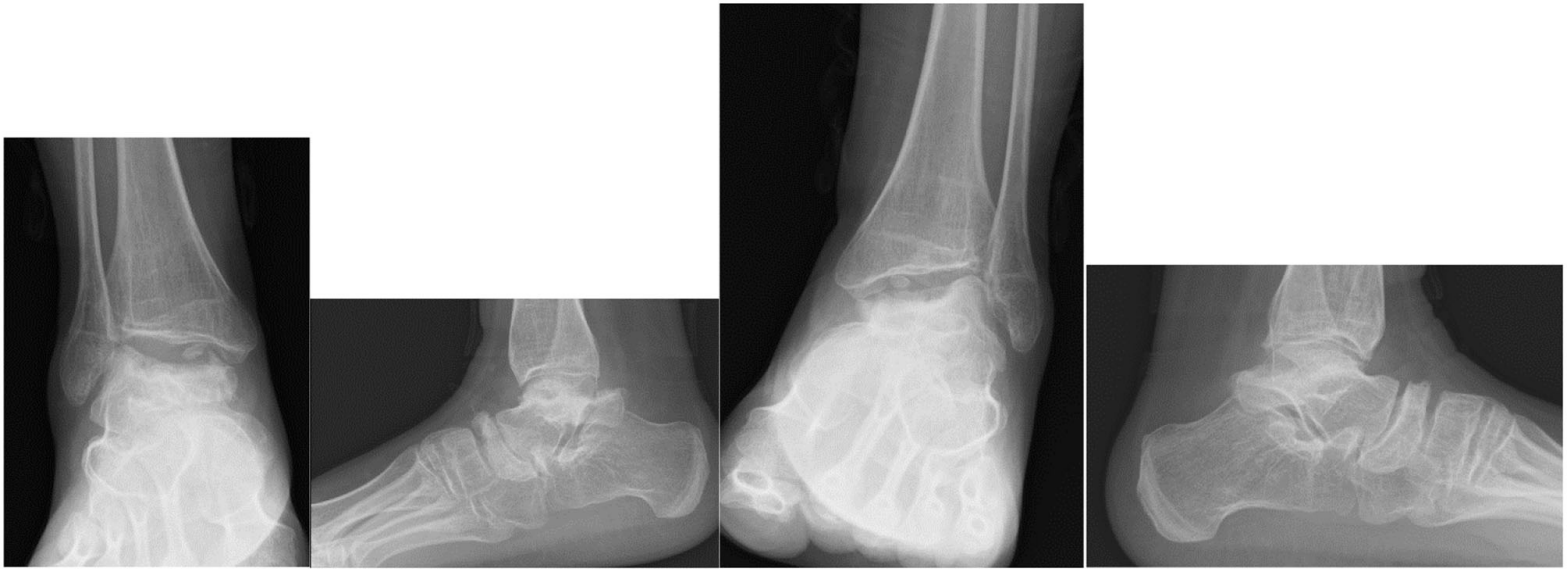
X-rays of both feet and ankles of a 20-year-old MPS IV patient with distal tibial epiphyseal dysplasia and valgus wedging of the tibial articular surface. Avascular necrosis, collapse and fragmentation of the talus and navicular can be seen bilaterally. Collapse of the 1st metatarsal head and arthritis of the 1st metatarso-phalangeal joint was also seen on the CT.

Dhawale et al. (178) a retrospective case series of 23 pediatric patients revealed ankle valgus, hip subluxation and genu valgum in all patients. Eight patients underwent operative correction of ankle valgus associated with lateral distal tibial epiphyseal dysplasia and fibular hypoplasia, with either Distal Tibial Osteotomy (DTO) (12 procedures) or Distal Tibial Hemiepiphysiodeses (DTH). The mean age at surgery was 11.1 years and the remaining potential for skeletal growth was likely causal in 4 ankles that had DTH, requiring revision surgery. Mean LDTA went from 70° pre surgery, to 80° degrees post surgery, but there was residual ankle valgus. Functional improvement was not reported. In an adult population DTH would not be appropriate, but DTO may be an appropriate operative intervention (168, 178, 188–190). Figure 10 demonstrates radiological changes in an adult patient with MPS IV.

**Figure 11:**
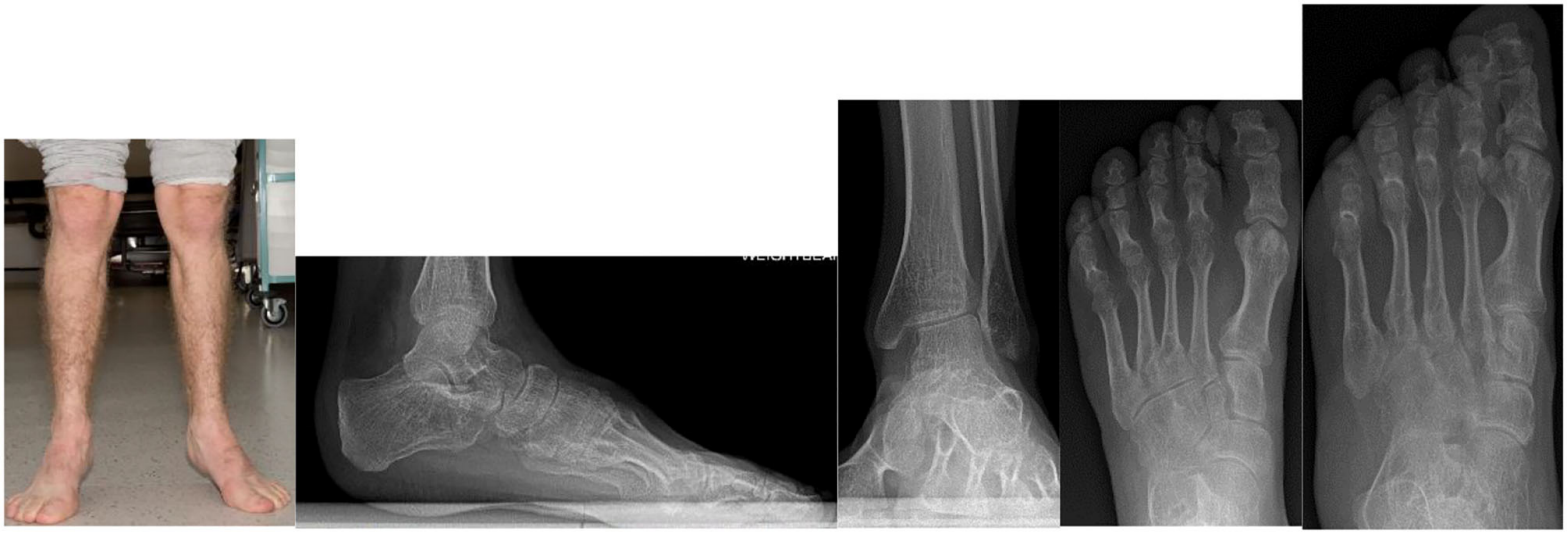
Clinical photograph of a 26- year- old male with MPS VI in fixed equinus contracture, caused by gastro-soleus spasticity secondary to spinal cord compression at the level of the conus medullaris. Radiographs of the left foot and ankle reveals distal tibial epiphyseal dysplasia with valgus wedging, decreased LDTA, syndesmotic widening with lateral talar shift and increased medial clear space suggestive of ligamentous deficiency, shortened “bullet” metatarsals and an hypoplastic intermediate cuneiform.

MPS VI (Maroteaux-Lamy syndrome) patients will have disproportionate, short trunked, short stature and commonly develop abnormalities of the spine including kyphosis, scoliosis and spinal canal compromise. Epiphyseal dysplasia leads to hip dysplasia and genu valgum (61, 191). In the foot and ankle TTS has been reported (181). Ankle equinus, muscle contractures, progressive decline of gait or spasticity must be investigated as potential signs of spinal canal compression. Figure 11 demonstrates the clinical and radiological changes seen in an adult patient with MPS VI.

MPS VII (Sly Syndrome) is a rare form of MPS with ataxia and seizures, and severe cases show progressive cognitive decline (119). Musculoskeletal deformities include dysostosis multiplex (including hypoplastic bones and bullet metatarsals), joint contractures, C1-2 instability from odontoid hypoplasia (possibly causing secondary spasticity) and bony dysplasia (171, 192).

Any MPS patient with foot and ankle problems should be referred to an orthopedic specialist with experience in this field, at a center that deals with adult MPS patients to ensure that the appropriate pathways and required members of the MDT are available.

Non-operative management of foot and ankle deformity should be exhausted prior to consideration of operative intervention, due to the complexity and risks of anesthesia (193–196). Orthotics are a mainstay of non-operative management of foot and ankle deformities in this group. Instability and laxity requires correction, splinting and supporting the foot in a more “normal,” plantigrade position. Stiffness and fixed deformity requires accommodation with insoles to fill the space between the floor and the foot to dissipate forces more evenly. Hindfoot valgus can be corrected (flexible deformity that can be corrected by hand) or accommodated (if deformity is not correctable by hand) with custom made insoles, AFO (Ankle-Foot-Orthoses) splints or custom made footwear. Equinus contractures can be accommodated with heel raise insoles, built up shoes or heeled shoes. Physiotherapy can improve function with muscle training and stretching of tight muscles and joints. Regular physiotherapy may slow musculoskeletal decline.

Botox can be useful in deformity related to spasticity from spinal cord compression as both a diagnostic test, a temporary therapeutic intervention and also to give specialists an idea of the possible outcome of surgery to lengthen or weaken a spastic muscle (197).

With limited literature on the outcomes of surgical intervention, especially in an adult population, and altered biology and behavior of bony and soft tissues, post-operative outcomes are unpredictable. An MDT approach improves pre-operative planning and reducing the number of surgeons performing operations in this cohort to as few specialists as possible for each body area can concentrate experience and shorten the learning curve.

TTS should be assessed with imaging to look for anatomical compression in the tarsal tunnel and nerve conduction tests. If a valgus hindfoot is present, correcting this may take the tension off the medially based nerve and relieve symptoms. Failing that, decompression may be beneficial.

Surgical correction of bony deformity may require corrective osteotomy (such as opening lateral wedge or closing medial wedge distal tibial osteotomy for ankle valgus) or fusion to realign the mechanical axis. Painful collapsed avascular necrosis may be treated with steroid joint injection, fusion or replacement.

Deformity caused by soft tissue laxity may be amenable to soft tissue reconstruction using synthetic material such as suture tape augmentation, to avoid recurrence. Although no evidence exists in this patient group, this has been used with excellent outcomes and safety in patients with soft tissue laxity from other etiologies (198). Joint fusion is another useful option.

Deformity caused by stiffness and contractures may be amenable to soft tissue release but recurrence has a high likelihood. Percutaneous release of flexor tendons to the toes, Achilles tendon lengthening or release of other soft tissues can improve function but does significantly weaken that function and could dramatically reduce function if done inappropriately.

For deformity caused by imbalance of the soft tissues, specialist assessment and gait analysis may be helpful to determine the cause, options and likely outcomes of surgery. Tendon or fascial lengthening, tendon release, tendon transfer and joint fusion are all options.

## Neurosurgical Issues

Neurosurgical manifestations of MPS vary per subtype affecting both cranial and spinal structures, and occur due to GAGs deposits in bone ossification centers, ligamentous tissue, facet capsules, arachnoid granulations and meninges (199).

For the purpose of this discussion, we will cover common spinal and cranial neurosurgical considerations in MPS, including pathogenesis, clinical manifestations, diagnosis and management (Table 6).

**Table 6:** MPS sub types and spinal disorders (200) – absence of feature, ± present in some cases, ±± present in majority of cases, ± ± ± present in all cases.

	**Eponym**	**Cervical Stenosis**	**Atlantoaxial instability**	**Thoraco-lumbar kyphosis**	**Scoliosis**
MPS1 (severe)	Hurler	++	+	++	+
MPS1 (attenuated)	Hurler-Scheie, Scheie	++	–	+	+
MPS II	Hunter	++	–	++	+
MPS III	Sanfilippo	–	–	+	+
MPS IV	Morpuio	+	+++	++	+
MPS VI	Maroteaux-Lamy	+++	+	++	+

### Spinal Manifestations in MPS

MPS spinal pathogenesis commences *in utero*, with most abnormalities clinically apparent in the early years, however, there these conditions need to be considered in the growing population of MPS patients surviving into adulthood.

**Figure 12:**
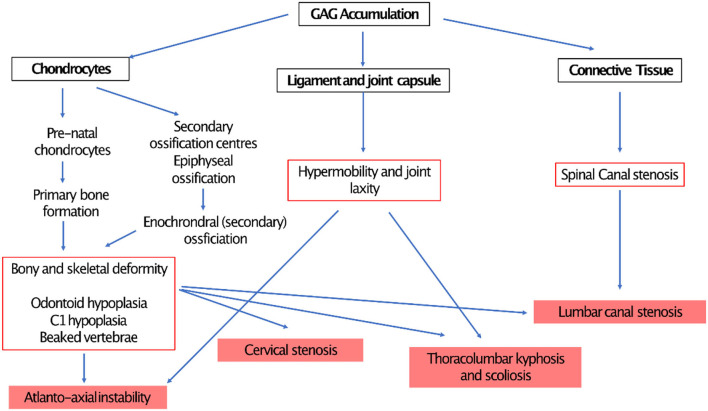
demonstrating pathophysiology of spinal compression in MPS (190).

Pre-natal accumulation of GAG in chondrocytes in primary ossification centers results in severe skeletal malformation, whilst secondary ossification and epiphyseal growth plate involvement leads to abnormal endochondral and membranous bone growth after birth, resulting in impaired growth. GAG deposits in ligaments and joint capsules induces inflammation and degeneration, causing either joint stiffness or hypermobility and ligamentous laxity (18). GAG accumulation in connective tissue, particularly within the anterior extra-dural space and ligamentum flavum contributes to spinal canal stenosis (Figure 12).

Although ERT and HSCT have proven beneficial in soft tissue manifestations of MPS, there is little evidence of success in bone or cartilaginous manifestations, hence the need for timely identification and intervention for these issues in order to protect neurological issues and prevent neurological deterioration (201).

Spinal manifestations of MPS include atlanto-axial instability, thoracolumbar kypho-scoliosis and cervico- and lumbar canal stenosis, with their severity depending on MPS type and level of disease activity (200). Most spinal MPS manifestations present in childhood, requiring surgical intervention to preserve neurological function. Surveillance imaging is recommended from 1 year old, even in the context of a normal neurological examination as MRI often identifies sub-clinical spinal cord injury (202, 203). With improved MPS therapies through ERT and HSCT and enhanced multidisciplinary care, more MPS patients are surviving in adulthood; it is imperative that this monitoring is continued beyond childhood as in some instances spinal cord compression may progress slowly, with surgical intervention only becoming necessary in adulthood (204, 205). Although studies specifically describing adult MPS spinal presentations are rare, the same constellation of pathologies affects adults and children. Cervical myelopathy with cord compression has been identified in 55% of adult MPS IVA patients (155), and those with slowly progressing MPS IV underwent primary cranio-cervical decompression and fixation at a mean age of 24 years (206). Furthermore, MPS patients who underwent surgical intervention during childhood often require further intervention as an adult, highlighting that MPS disease status can remain dynamic despite advancements in medical therapies and early surgical intervention (18, 207). From a technical surgical perspective, surgical fixation can induce “adjacent segment disease,” in which spinal fixation causes increased biomechanical stress through neighboring spinal segments, accelerating degenerative changes often leading to further surgery (208, 209). We should therefore anticipate that the ligament, cartilaginous and bony abnormalities in MPS adults would predispose to earlier onset adjacent segment disease and the possibility of rapid neurosurgical deterioration.

### Atlanto-Axial Instability (AAI)

**Figure 13:**
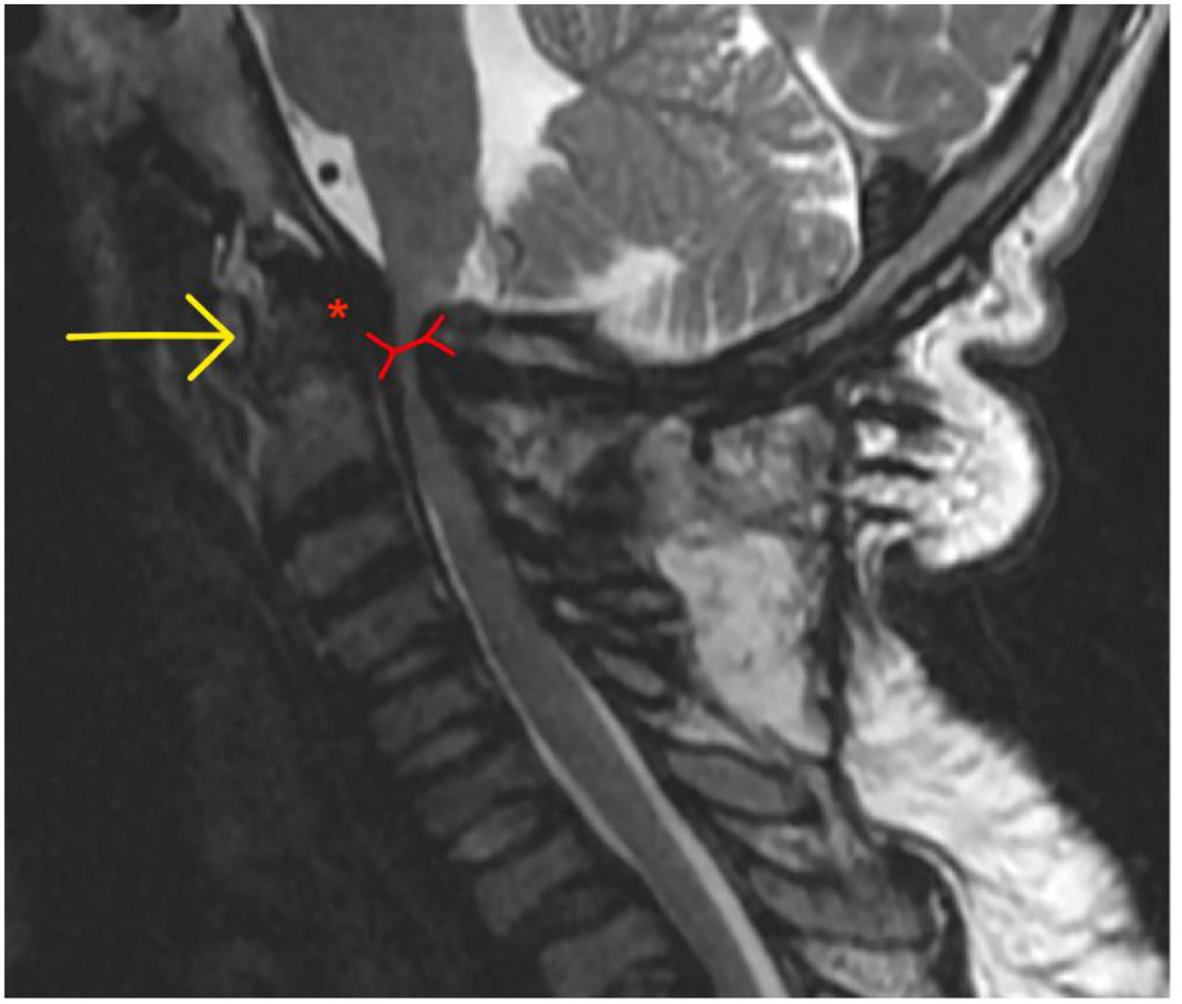
Atlanto-axial instability in adult MPS VI adult MPS VI patient demonstrating atlanto-axial instability with hypoplasia of the dens (yellow arrow), reduction in spinal canal dimensions between posterior body of C2 and posterior arch of C (red arrow), exacerbated by GAG accumulation in the anterior spinal space (red asterix).

AAI (MPS I, IV, and VI) is caused by the trifecta of skeletal deformity (hypoplastic dens and posterior arch of C1), ligamentous hyper mobility and canal stenosis due to extra dural GAG accumulation. This results in repeated impingement of C2 and the posterior arch of C1 during flexion and extension causing irreversible spinal injury (202, 210) (Figure 13).

Repeated injury to the high cervical cord can result in progressive cervical myelopathy, clinically identified by upper motor neurone findings (lower extremities > upper extremities) with a spastic paraparesis of lower limbs, abnormal gait, hyperreflexia, clonus and Hoffman's positive. At its most severe, and in delayed presentation, AAI can present as quadriparesis and respiratory failure (190).

Patients may report numb fingertips and difficulty with fine manual dexterity (buttons, zips, picking small items up from a table), plus deterioration in gait (balance and endurance); in more gradual onset disease or those surviving beyond childhood, these symptoms may present slowly over years and be attributed to non-neurological MPS causes and learning difficulties, again demonstrating the need for surveillance imaging regimens in patients who do not undergo surgical fixation in childhood.

AAI is also associated with axial neck pain; this is less likely to occur in MPS cervical stenosis.

Flexion and extension radiographs identify AAI by assessing the distance between posterior wall of the odontoid process and anterior wall of posterior arch of C1 (211), whilst CT scans provide 3-dimensional characterization of odontoid hypoplasia. MRI delineates the cervical cord compression, with hyperintense T2 signal change representing myelomalacia due to compression and cord injury. MRI is performed in a neutral spinal position, so may underestimate the extent of cord compression (212). If available, dynamic flexion/extension MRIs provide more accurate assessment of cord compression.

Optimum timing of surgery is a careful balance of timely decompression and fixation surgery before irreversible spinal cord damage and neurological injury occurs, and the technical difficulties of operating on immature vertebrae. Bony union of cervical vertebrae occurs between 3 and 6 years, so it is recommended that surgery is performed as close to 6 years old as possible (213). Surgical technique selected depends on individual anatomy and surgeon preference. Atlanto-axial fixation with either trans-articular or laminar screws or wiring is performed where there is sufficient atlas antero-posterior diameter and bulk, and is preferred as it preserves occipito-atlanto movement (214, 215). Occipital- cervical fusion is performed in instances where a C1 laminectomy is required to decompress the cord. Preoperative imaging to assess configuration of vertebral arteries should be performed in all instances (216). These techniques are utilized in both pediatric and adult cases of MPS induced AAI.

### Cervical Stenosis

**Figure 14:**
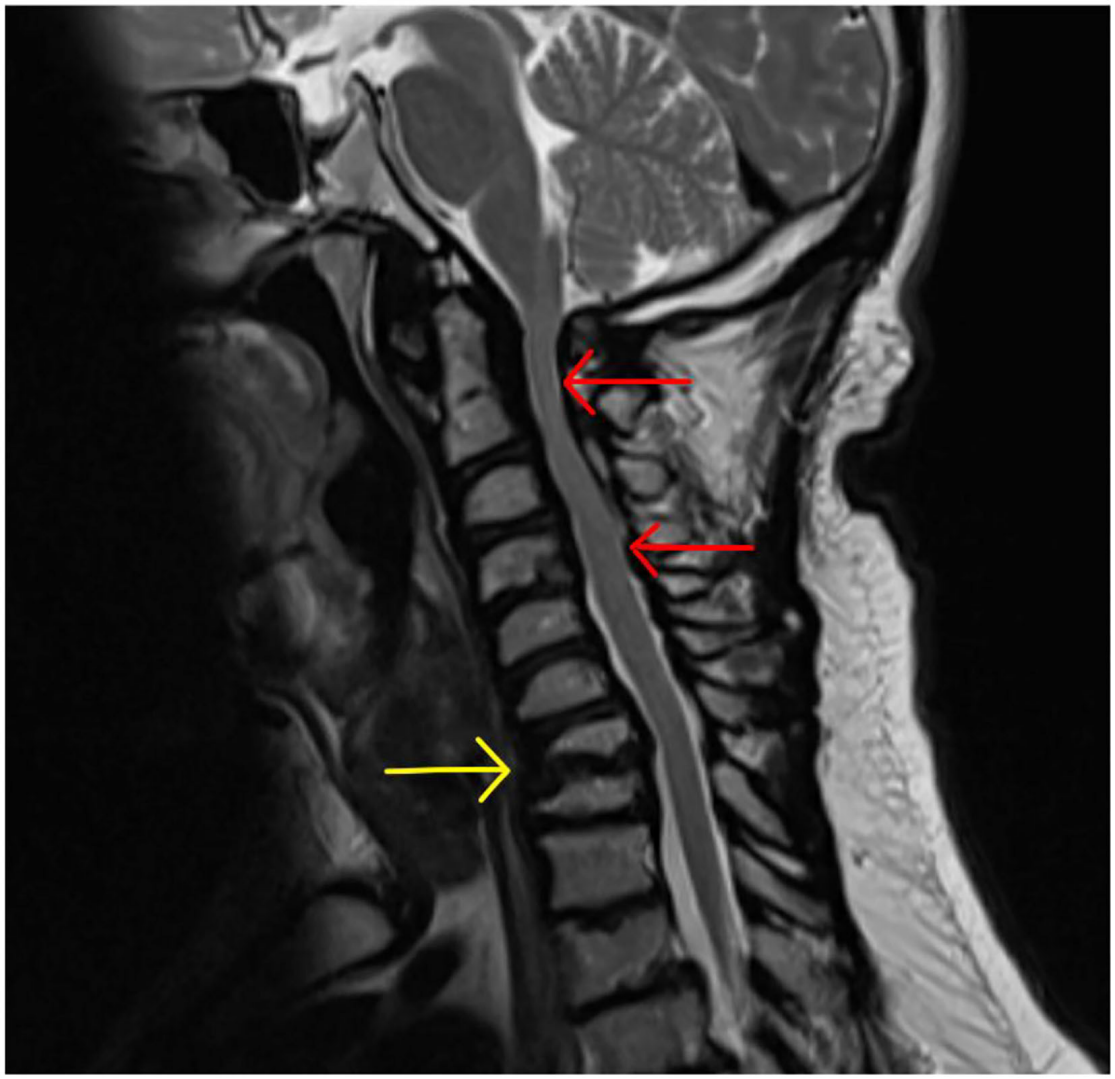
Cervical cord stenosis. Sagittal MRI of adult with MPS I demonstrating cervical cord stenosis at C2–C4 and hypertrophied ligament, with effacement of anterior and posterior CSF spaces (red arrows). Also note the abnormal vertebrae at C7 and T1 with degenerative disc and hypertrophied ligament (yellow arrow) causing cervical-thoracic kyphosis and effacement of anterior CSF spaces.

Cervical stenosis the most commonly occurring spinal pathology in MPS patients (MPS I, II, VI) and occurs due to accumulation of GAG in epidural connective tissue and ligamentum flavum plus the additional impact of hypoplastic posterior C1 arch and degenerative facet joints and intervertebral discs (207) (Figure 14).

Clinical manifestations depend on the anatomical level and severity of compression, but typically presents with numb, clumsy hands and difficulty with fine motor tasks. Carpal tunnel syndrome should be excluded in these instances, as this is also common in MPS I, II and III (217). Delayed presentation of cervical stenosis would present with a progressive myelopathy as in AAI (above), and has been reported in. 55% of adult MPS IVA patients (155).

Diagnoses is achieved by MRI, identifying a narrowed cervical canal with effacement of CSF spaces, hypertrophied ligamentum flavum and degenerate intervertebral discs. High T2 signal within the spinal cord confirms potentially irreversible injury.

As with AAI, surgical intervention should occur before permanent neurological disability occurs. In the absence of cervical spine instability, posterior decompression in the form of cervical laminectomy or laminoplasty is appropriate. Laminoplasty avoids the potential complications of subsequent instability, and dural fibrosis (202). Halo vests are often applied post laminectomy to stabilize the neck in the immediate post-operative period. As with AAI approaches, these surgical approaches are applicable to all MPS age groups.

### Thoracolumbar Kyphosis

**Figure 15:**
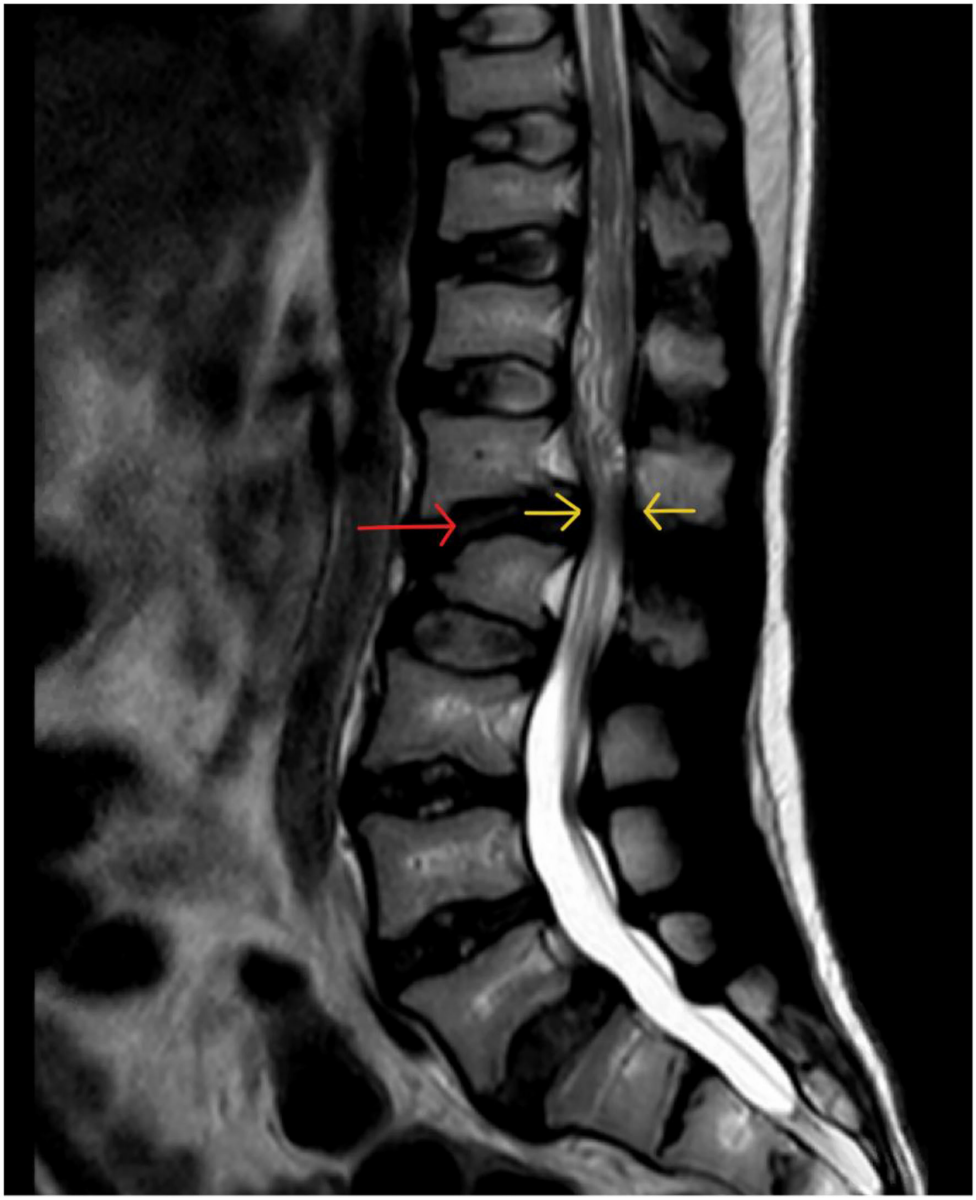
Thoraco-lumbar kyphosis. T2-weighted sagittal MRI of the adult spine with MPS VI, demonstrating abnormal vertebra body shape at L2, resulting in kyphosis at L1-L2 (red arrow) and early spinal canal compromise (yellow arrows).

Thoracolumbar kyphosis occurs due to bony deformity at the thoraco-lumbar junction due to incomplete endochondral ossification, and is often accompanied by scoliosis. The progressive bony deformity results in loss of sagittal balance and a compensatory thoracic lordosis, pelvic anteversion and hip and knee flexion to maintain standing balance (218) (Figure 15).

Patients typically present with a visible spinal deformity that is monitored *via* surveillance xrays. Neurological deterioration is a widely accepted indication for surgical intervention, or when the Cobb angle is over 40°, however there is little consensus otherwise, with good evidence supporting the use of sequential bracing once a patient is able to maintain sitting position (218, 219). Surgical fixation often includes vertebrectomy (to remove the deformed vertebrae), followed by posterior fixation with pedicle screws and rods (219), and is usually performed between ages 8 and 13 years. In adult patients with MPS, surgery is reserved in instances of sudden, severe neurological deficits or extreme kyphosis (220).

### Lumbar Canal Stenosis (LCS)

LCS in MPS is less common than cervical spine pathologies, however should be considered in MPS patients who present with LCS symptoms, typically at a younger age than the non-MPS population (>70 years). Similar to cervical cord stenosis, LCS occurs due to GAG accumulation in ligaments and soft tissue, aggravated by facet join hypertrophy and disc degeneration, as well as MPS related bony deformity due to scalloping and beaking of bone edges (221). LCS in MPS will present as the non-MPS population, with lower back pain, lower limb claudicant pain and bladder or bowel dysfunction, although it should be noted that claudicant pain will only be detected in MPS patients able to mobilize for a prolonged period (18). LCS should therefore be considered in non- and minimally-ambulant MPS patients with pain and altered sphincter function as they progress through adulthood. Decompressive surgery in the form of lumbar laminectomy is indicated in the event of severe leg pain and bladder and bowel disturbances. Spinal fusion would be indicated in the event of degenerative changes leading to an unstable spondylolisthesis.

### Hydrocephalus

Communicating hydrocephalus is an early manifestation in MPS, most commonly occurring in MPS I and II. CSF absorption is impeded by GAG accumulation in arachnoid granulations and at meningeal CSF absorption sites, further confounded by reduced venous drainage due to skull base abnormalities (200, 222). Accumulation of CSF leads to ventriculomegaly, raised intracranial pressure and in infants, macrocephaly. As in the non-MPS population, hydrocephalus presents with progressive headache, nausea, vomiting, and reduced visual acuity due to sustained optic nerve compression. Presentation in MPS patients may be insidious due to accompanying brain atrophy, again advocating the need for surveillance regimens, and vigilance for gradually progressive onset symptoms in adult patients.

MRI brain and CSF flow studies will confirm ventriculomegaly and communicating hydrocephalus, whilst the Evan's index helps distinguish true hydrocephalus from brain atrophy and hydrocephalus ex-vacuo (223).

Surgical management of hydrocephalus in MPS is aimed at establishing permanent CSF diversion and reducing the effects of sustained elevated ICP on the brain. Typically, this is achieved through the insertion of a ventriculoperitoneal shunt. Although a routine neurosurgical procedure, performed in both adults and children, shunt insertion in MPS is associated with a higher rate of blockage (likely due to GAG accumulation) and subsequent revision procedures than the non-MPS population, plus a higher incidence of intracranial hemorrhagic complications (224).

### General Anesthetic Considerations

General anesthesia in MPS is complicated by many factors, and should be performed by anesthetic teams experienced with MPS patients. Anatomically, MPS patients often have hypertrophied tonsils and epiglottis plus macroglossia, often leading to a challenging intubation exacerbated by the limited neck movement permitted during to intubation in order to avoid exacerbating cervical cord injuries. Furthermore, the short stature and neck seen in many MPS patients makes prone positioning challenging (225).

## Bone and Cartilage

Skeletal abnormalities in MPS disorders result from GAG accumulation in chondrocytes. The process probably starts *in utero* (226). In MPS IVA keratan sulfate accumulation disturbs bone mass development and perturbs the regular microarchitecture of bone tissue in terms of trabecular or cortical thickness (1) and affects growth plate functioning (138, 227). GAG accumulation directly affects joints through activation of the Toll Like Receptor 4 (228, 229) and results in short stature. The latter results from a combination of joint contracture, bone growth plate disorganization and failure, and endocrine abnormalities (127, 230). Endocrine gland dysfunction (127) or nutritional deficiencies can also cause growth failure in MPS patients. Adults with attenuated MPS types have atypical clinical symptoms including normal stature.

The literature describing the effects of ERT on bone mineral density (BMD) in MPS is limited (130, 231, 232). Several possible mechanisms for the improvement of BMD in MPS patients receiving ERT need to be considered. Reduced GAG storage in the bones, increased muscle strength and endurance, and improved pulmonary function resulting in improved mobility, are possible explanations (74, 233). There is a clinical need for sensitive and MPS specific bone biomarkers in these conditions to monitor their response to therapy efficiently, e.g., plasma interleukin-6 and urine pyridinolines (234).

DXA proves to be a clinically useful method for monitoring BMD in MPS patients and assessing its response to ERT (232). It is challenging to accurately interpret DXA results when accessing bone health in MPS patients, given their considerable height deficits, relatively thick skulls, and macrocephaly. Therefore, BMD *z*-scores need to be adjusted for height-for-age *z*-score according to the method of Zemel et al. (235) that was recommended to detect bone mass in MPS patients (130, 233, 236).

A high prevalence of osteopenia (31%) or osteoporosis (15%) has been documented in MPS I, II, and VI patients under 19 years of age (232) and MPS III (237) which may be further compounded by age-related decrease in BMD in adult MPS patients. In addition, nutritional deficits, e.g., vitamin D deficiency increases the risk of fractures in older and immobile patients (237).

## Endocrine

Metabolic syndrome is an uncommon long-term complication of HSCT in MPS IH (238). In one study, 53% of the patients had one or more metabolic syndrome components; raised blood pressure, raised serum triglycerides, central obesity, low high-density lipoprotein (HDL) cholesterol or elevated fasting blood glucose. These isolated components occurred irrespective of the patients' previous radiation prior to HSCT (238). Another study has shown that adult MPS IH patients do not often develop significant hypercholesterolaemia or diabetes mellitus despite an increased BMI (239). Insulin resistance and diabetes mellitus have been previously observed but resolved after the introduction of lifestyle modifications (239).

Thyroid gland abnormalities have been documented in pediatric (127) and adult MPS IH patients post HSCT (240), with patients experiencing clinical or subclinical hypothyroidism thyroiditis or hyperthyroidism.

Although in pediatric patients younger than 10 years of age, there were no cases diagnosed with gonadal failure (127), hypergonadotrophic hypogonadism has been documented in the adult population (241). Endocrine abnormalities are common in children and adults who received HSCT, in particular those who were exposed to total body irradiation (127). Premature adrenarche, precocious puberty and primary amenorrhea, premature ovarian insufficiency have been previously observed in this cohort of patients (127, 240). Despite the hormonal abnormalities, pregnancy was described in a female with MPS IH post HSCT (242).

Impaired growth is a feature of MPS disorders regardless of therapy. Children and adults with MPS IH post HSCT have short stature (127, 230, 243) but it is difficult to say whether it results purely from the hormonal dysfunction post HSCT or whether the effects innate to MPS IH play a role as well.

## Hematology

Bone marrow involvement is a feature of LSDs and manifests with anemia, leukopenia, thrombocytopenia or bicytopenia/pancytopenia. Thrombocytopenia is more common in LSDs, whereas anemia and leukopenia are observed less frequently. Whereas nasal and gingival bleeding are common in patients with severe thrombocytopenia; mild anemia with no pancytopenia can remain undiagnosed.

MPS disorders manifest with hypersplenism, leading to pancytopaenia. It has been described in an MPS IIIB case who also presented with hypocellular bone marrow (244). In another adult case with MPS IIIB, moderate neuropaenia was observed in the context of mild hypersplenism. In view of the lack of symptoms, the diagnosis was incidental.

In MPS II idiopathic thrombocytopenic purpura (ITP) was documented (245, 246). In two other MPS II siblings it was accompanied by pancytopenia after ERT (247).

## Gastrointestinal Manifestations

Episodic or recurrent diarrhea has been documented in 50–92% of patients with MPS IIIA, B, and C (248–250) interspersed with bouts of constipation, frequently present in older patients (249). In MPS III adults with severe neurocognitive dysfunction, swallowing problems may become apparent, often requiring gastrostomy (251).

In two described MPS IIIB cases (252, 253) chronic diarrhea and endoscopic and histological findings were compatible with intestinal lymphangiectasia; dilated lymphatic vessels in the lamina propria, without evidence of inflammation. In both cases, their symptoms improved after a dietary treatment with a low-fat diet supplemented with medium-chain triglyceride.

The pathophysiology of these symptoms in MPS III is not known, with fecal calprotectin often remaining within the normal range (252). It was hypothesized that localized heparan sulfate deposits may cause a mechanical obstruction of intestinal lymphatics that may lead to fluid loss and diarrhea. It was consistent with the dilated lymphatic vessels in the ileal lamina propria and abnormal pattern on scintigraphy with Tc-99m–labeled human serum albumin (252).

Heparan sulfate is not only an important component of the extracellular matrix and cell surface but it is involved in important cell functions (cell motility, interaction between the intra- and extracellular matrices) and in regulation of extracellular signaling pathways (e.g., by modulating fibroblast growth factor cytokine activity) (252, 254, 255). Heparan sulfate has a role in protein-losing enteropathy, with correlations with protein leakage and modulation of cytokines, although the molecular mechanisms that mediate this effect are unknown (254, 255).

Gastrointestinal findings with recurrent and occasionally severe diarrhea are described in MPS II (3, 256). Loss of bowel control and fecal incontinence were also reported.

Adult MPS type IH post HSCT may present with irritable bowel syndrome-like symptoms. It was postulated that their long-term symptoms might have been attributed to previous total body irradiation in childhood. Patients with other MPS types (i.e., MPS IVA) regardless of the treatment status, may also suffer from persistent diarrhea and abdominal discomfort.

## Skin Problems

Non-specific cutaneous lesions can be seen in all MPS types. Thickened skin with a loss of elasticity on elbows and knees, rough hair, hypertrichosis, and generalized hirsutism are common features.

In MPS II, ivory-colored papules and nodules coalesce into a reticular pattern resembling irregular cobblestones or pebbles over the scapular region and outer lateral sides of the arms and thighs. The lesions usually present before the age of 10 years and may spontaneously regress (257). Histopathology investigations shows irregularly organized collagen bundles separated by interfibrillar substance (258). Their response to available therapies has not been investigated in clinical trials, but given that it is not commonly seen in adult MPS II patients on ERT, it raises a question whether the papules improve with treatment.

Thickened skin over the hands is often observed in MPS I and II patients, even in attenuated MPS types presenting in adolescence or adulthood. The skin tightening and thickening can lead to contractures of the fingers which affects the manual dexterity.

Dermal melanocytosis (Mongolian spots) is common in infancy, particular in infants of Asian, Native American, and African descent. Mongolian spots are found in children with MPS, particularly with Hurler syndrome (259, 260) and persist into adulthood, and often continue to progress over time (259, 260). The lesions are thought to result from an interruption of the migration of pigmented melanocytes from the neural crest to the epidermis (259). In MPS, however, they may be due to the accumulation of stored heparan sulfate, which are tightly associated with high-affinity tyrosine kinase–type receptor for nerve growth factor (260).

Extensive telangiectasias have been previously described in MPS IV (261). Folliculitis-like lesions in treatment naive MPS IVA adult patient have been thought to be attributed to keratan sulfate in the skin of the arms and back (Table 7).

**Table 7:** Summary of the common systems affected with symptoms and signs.

**System**	**Common symptoms**	**Signs**
Upper and central airways	Breathlessness Poor exercise tolerance Snoring and sleep apnoea Stridor Swallowing problems Voice changes	Bulky oropharynx, larynx, hypopharynx, airway narrowing, tracheal stenosis or malacia or tracheal tortuosity, extrinsic compression of the trachea, poor dentition, poor laryngeal elevation GAG deposition and bulky soft tissues of upper and lower airways
Respiratory	Cough Mucous Sleep apnoea Breathlessness Poor exercise tolerance	Adopting sniffing position, hypoxia, tracheobronchomalacia, restrictive lung functions, mucus retention, central/peripheral sleep apnoea, cardiorespiratory failure, Chronic infections
Neuropsychology	Behavioral problems Cognitive decline Poor social and interpersonal skills, ataxia, seizures	Poor compliance in home and with carers, anxiety, fear, low self-esteem, depression, social, withdrawal, self-harm, at risk of exploitation, safe guarding issues, caregiver burden
Pain	Pain Restriction in physical activity Angina	Local tenderness, bone and soft tissue deformity joint stiffness, contractures, cardiac causes Spinal canal stenosis, myelopathy, atlanto axial instability
Foot and ankle	Pain Gait and walking problems deformity	Short stature, skeletal dysplasia, contractures Pes planus, ankle valgus, distal tibial wedging, shortened fibula, equinus, cavovarus, tarsal tunnel syndrome, curly toes, ligamentous laxity and hypermobility in MPS IV, avascular necrosis
Neurosurgical	Pain Deformity Mobility issues	Skeletal malformation, atlantoaxial instability, kyphoscoliosis cervico-lumbar canal stenosis, myelopathy and cord compression, hydrocephalus
Endocrine	Short stature High weight Non-specific symptoms	Central obesity, hypertension, hypercholesterolemia, vitamin D deficiency Hyperglycemia, high body mass index, insulin resistance, hypo or hyper thyroidism, gonadal failure, premature adrenarche, precocious puberty, primary amenorrhoea, ovarian failure
Hematological	Tiredness Fatigue Poor exercise tolerance	Anemia, leukopenia, thrombocytopenia, hypersplenism, pancytopenia, neutropenia, idiopathic thrombocytopenic purpura
Gastrointestinal	Swallowing problems, constipation, diarrhea, loss of bowel control, fecal incontinence, abdominal discomfort	Dysmotility, poor oropharyngeal control Intestinal lymphangiectasia, organ dysfunction at microscopic and macroscopic levels
Skin	Thickening lesions, stiffness	Thickened skin, loss of elasticity, coarse features, hypertrichosis, hirsutism, nodules, mongolian spots, telangiectasia

## Conclusions

Diagnostic and therapeutic developments lead to improved longevity in adult MPS patients. Multisystemic presentation causes a variety of symptoms including neurocognitive dysfunction, chronic pain and poor quality of life. Lack of understanding due to the rarity of the disease and a lack of formal diagnostic and therapeutic pathways impose clinical challenges. The progressive nature of the disease and lack of disease specific biomarkers for adult MPS make it impossible to monitor disease progression. It mandates long-term follow-up care by various specialists preferably with a special interest in adult MPS in specialist tertiary metabolic centers. This multisystem disease warrants a multi-professional approach including physicians, surgeons and allied healthcare professionals such a physiotherapists, psychologists, speech and language therapists, specialist nurses, dieticians. The adult metabolic physician plays a central role in leading the management of these complex patients and co-ordinating with all the specialities. Sharing knowledge of MPS with various health professionals and working closely with patient support groups is important in the holistic care of adult MPS patients. Improving knowledge and raising awareness of adult MPS amongst various healthcare professionals will help identify the attenuated phenotypes of MPS, which often present with atypical symptoms in adulthood. The impact of an MPS diagnosis affects not only the patient but their family, hence necessary family support should be considered where possible. The onus is on a multispecialty and multicenter approach to collaborate, share experience and address the increasing need for formal clinical guidelines and pathways in management of this complex multisystem disease.

## Author Contributions

KS and CG: conceptualization, validation, resources, writing, and editing. KS, AB, CC, MD, PO, CP, AS, JR, EG, GT, and CG: resources, writing, data curation, methodology, and writing original draft. All authors have read and approved the final manuscript.

## Funding

The authors declare that this research received no external funding. The article processing fee was supported by BioMarin Pharmaceutical Inc. The funder was not involved in the study design, collection, analysis, interpretation of data, the writing of this article or the decision to submit it for publication.

## Conflict of Interest

The authors declare that the research was conducted in the absence of any commercial or financial relationships that could be construed as a potential conflict of interest.

## Publisher's Note

All claims expressed in this article are solely those of the authors and do not necessarily represent those of their affiliated organizations, or those of the publisher, the editors and the reviewers. Any product that may be evaluated in this article, or claim that may be made by its manufacturer, is not guaranteed or endorsed by the publisher.

## Abbreviations

ADLs: activities of daily living
AFO: Ankle-Foot-Orthoses
BMT: Bone Marrow Transplantation
CHQ-PF50: Child Health Questionnaire-Parent Form 50
CSA: central Sleep Apnea
CLD: chronic lung disease
CNS: central nervous system
CPAP: continuous positive airway pressure
DAMPS: Damage-associated Molecular patterns
DTO: Distal Tibial Osteotomy
DTH: Distal Tibial Hemiepiphysiodeses
ERT: Enzyme Replacement Therapy
FLACC, Face, Legs, Activity, Cry: Consolability scale
FRC: functional residual capacity
GAGs: glycosaminoglycans
HR-QoL: health-related quality of life
HSCT: Hematopoietic Stem Cell Transplantation
IL-1β: Interleukin 1 Beta
LDTA: Average lateral distal tibial angle
LPS: Lipopolysaccharide
LSD: lysosomal storage disorder
MDT: Multidisciplinary team
MEP: maximum expiratory pressure
MIP: maximum inspiratory pressure
MMPs: Matrix Metalloproteinases
MPS: Mucopolysaccharidosis
MPS IH: Hurler syndrome
MPS IH/S: Hurler-Scheie
MPS IS: Scheie
NIV: non-invasive ventilation
OSA: obstructive sleep apnoea
OSAS: obstructive sleep apnoea syndrome
PAP: positive airway pressure
PH: pulmonary hypertension
RANKL: Receptor Activator of Nuclear factor Kappa- β Ligand
SCT: stem cell transplant
SDB: sleep disordered breathing
SNIP: sniff nasal inspiratory pressure
SRT: substrate reduction therapy
TBM: tracheobronchomalacia
UA: upper airways
UARS: upper airway resistance syndrome
TC: Talo-calcaneal
TM: tracheomalacia
TNF-α: Tumor Necrosis Factor Alpha
TLR4: Toll like receptor 4
TTS: Tarsal tunnel syndrome.
