# Supplementary material for: Non-cardiac Manifestations in Adult Patients With Mucopolysaccharidosis
Source: Front Cardiovasc Med. 2022 Mar 7;9:839391. doi: 10.3389/fcvm.2022.839391 (PMC8935042; doi:10.3389/fcvm.2022.839391)
Supplement: Supplementary file 1 [file Data_Sheet_1.docx]

Appendix: Salford Mucopolysacharridosis Airway Score (SMAS); reproduced from Gadepalli et al (30)

| **Serial number** | **Parameter** | **Measure** | **Score** | **Final Score** |
| --- | --- | --- | --- | --- |
|  | **MPS type** |  |  |  |
|  | **Mouth opening** | >5cm | 0 |  |
|  |  | 4-5cm | 1 |  |
|  |  | 3-4cm | 2 |  |
|  |  | <3cm | 3 |  |
|  |  |  |  |  |
| **2** | **Teeth protrusion on clinical exam and scans** | Non-protruding | 0 |  |
|  |  | Mild | 1 |  |
|  |  | Moderate | 2 |  |
|  |  | Severe | 3 |  |
|  |  |  |  |  |
| **3** | **Cervical spine mobility, stability** | unrestricted | 0 |  |
|  |  | 60-90 degrees flexion | 1 |  |
|  |  | 30-60 degrees flexion | 2 |  |
|  |  | < 30 degrees or unstable | 3 |  |
|  |  |  |  |  |
| **4** | **Tongue bulkiness on examination and Scan** | Normal | 0 |  |
|  |  | Mild | 1 |  |
|  |  | (Filling less than 1/3 of floor mouth) |  |  |
|  |  | Moderate | 2 |  |
|  |  | (Filling 1/3 to 1/2 of oral cavity) |  |  |
|  |  | Severe | 3 |  |
|  |  | (Filling more than 1/2 of oral cavity) |  |  |
|  |  |  |  |  |
| **5** | **Modified Mallampati grade [19]** | 1 | 0 |  |
|  |  | 2 | 1 |  |
|  |  | 3 | 2 |  |
|  |  | 4 | 3 |  |
|  |  |  |  |  |
| **6** | **Thyromental distance** | >6 cm | 0 |  |
|  |  | 5-6 cm | 1 |  |
|  |  | 4-5 cm | 2 |  |
|  |  | <4 cm | 3 |  |
|  |  |  |  |  |
| **7** | **Larynx height epiglottis to soft palate** | >4cm | 0 |  |
|  |  | 3-4cm | 1 |  |
|  |  | 2-3cm | 2 |  |
|  |  | <2cm | 3 |  |
|  |  |  |  |  |
| **8** | **Epiglottis bulkiness** | Normal | 0 |  |
|  |  | (Filling less than 1/3 of oropharynx) |  |  |
|  |  | Mild | 1 |  |
|  |  | (Filling 1/3 to 1/2 of oropharynx) |  |  |
|  |  | Moderate | 2 |  |
|  |  | (Filling 1/2 to complete oropharynx) |  |  |
|  |  | Severe | 3 |  |
|  |  | (Filling entire oropharynx) |  |  |
|  |  |  |  |  |
| **9** | **Supraglottis bulkines** | Normal | 0 |  |
|  |  | (Filling less than 1/3 of laryngopharynx) |  |  |
|  |  | Mild | 1 |  |
|  |  | (Filling 1/3 to ½ of laryngopharynx) |  |  |
|  |  | Moderate | 2 |  |
|  |  | (Filling ½ to complete laryngopharynx) |  |  |
|  |  | Severe | 3 |  |
|  |  | (Filling entire oropharynx) |  |  |
|  |  |  |  |  |
| **10** | **Glottis bulkiness** | Normal | 0 |  |
|  |  | (Filling less than 1/3 of glottis) |  |  |
|  |  | Mild | 1 |  |
|  |  | (Filling 1/3 to 1/2 of glottis) |  |  |
|  |  | Moderate | 2 |  |
|  |  | (Filling 1/2 to complete glottis) |  |  |
|  |  | Severe | 3 |  |
|  |  | (Filling entire glottis) |  |  |
|  |  |  |  |  |
| **11** | **Sub glottis diameter at cricoid level** | >7mm | 0 |  |
|  |  | 6-7mm | 1 |  |
|  |  | 5-6mm | 2 |  |
|  |  | <5mm | 3 |  |
|  |  |  |  |  |
| **12** | **Tracheo – malacia or tracheal stenosis** | No narrowing | 0 |  |
|  | **(degree of narrowing)** |  |  |  |
|  |  | 50-75% lumen narrowing | 1 |  |
|  |  | 75-99% lumen narrowing | 2 |  |
|  |  | 100% lumen narrowing | 3 |  |
|  |  |  |  |  |
| **13** | **Tracheal tortuosity** | None | 0 |  |
|  |  |  |  |  |
|  |  |  |  |  |
|  |  | present | 3 |  |
|  |  |  |  |  |
| **14** | **FEV1%** | >80% | 0 |  |
|  |  | 60-79% | 1 |  |
|  |  | 40-59% | 2 |  |
|  |  | <40% | 3 |  |
|  |  |  |  |  |
| **15** | **FVC%** | >80% | 0 |  |
|  |  | 60-79% | 1 |  |
|  |  | 40-59% | 2 |  |
|  |  | <40% | 3 |  |
